# From Bush Medicine to Modern Phytopharmaceutical: A Bibliographic Review of Devil’s Claw (*Harpagophytum* spp.)

**DOI:** 10.3390/ph14080726

**Published:** 2021-07-27

**Authors:** Thomas Brendler

**Affiliations:** 1 Department of Botany and Plant Biotechnology, University of Johannesburg, Auckland Park, Johannesburg 2006, South Africa; txb@plantaphile.eu; 2 Plantaphile, Collingswood, NJ 08108, USA

**Keywords:** *Harpagophytum*, devil’s claw, teufelskralle, grapple plant, sengaparile, harpagoside, nomenclature, ethnobotany, traditional use, trade, biochemistry, pharmacology, clinical, safety, toxicology, veterinary, review

## Abstract

Devil’s claw (*Harpagophytum* spp., Pedaliaceae) is one of the best-documented phytomedicines. Its mode of action is largely elucidated, and its efficacy and excellent safety profile have been demonstrated in a long list of clinical investigations. The author conducted a bibliographic review which not only included peer-reviewed papers published in scientific journals but also a vast amount of grey literature, such as theses and reports initiated by governmental as well as non-governmental organizations, thus allowing for a more holistic presentation of the available evidence. Close to 700 sources published over the course of two centuries were identified, confirmed, and cataloged. The purpose of the review is three-fold: to trace the historical milestones in devil’s claw becoming a modern herbal medicine, to point out gaps in the seemingly all-encompassing body of research, and to provide the reader with a reliable and comprehensive bibliography. The review covers aspects of ethnobotany, taxonomy, history of product development and commercialization, chemistry, pharmacology, toxicology, as well as clinical efficacy and safety. It is concluded that three areas stand out in need of further investigation. The taxonomical assessment of the genus is outdated and lacking. A revision is needed to account for intra- and inter-specific, geographical, and chemo-taxonomical variation, including variation in composition. Further research is needed to conclusively elucidate the active compound(s). Confounded by early substitution, intermixture, and blending, it has yet to be demonstrated beyond a reasonable doubt that both (or all) *Harpagophytum* spp. are equally (and interchangeably) safe and efficacious in clinical practice.

## 1. Introduction

Devil’s claw is the collective name of plants from the genus *Harpagophytum* (Pedaliaceae). The latter includes two species, *H. procumbens* (Burch.) DC. ex Meisn. and *H. zeyheri* Decne., currently divided into five subspecies with introgression reported from overlapping habitats [1,2]. The secondary root tubers of devil’s claw are used in botanical drugs and supplements and are exported from Southern Africa, mainly Namibia. Entrepreneurial spirit, colonialism, and the absence of regulatory barriers drove the commercialization of devil’s claw in a fashion similar to that of other medicinal plants from Southern Africa, such as Umckaloabo (*Pelargonium sidoides*) [3], rooibos (*Aspalathus linearis*) and honeybush (*Cyclopia* spp.) [4], buchu (*Agathosma betulina*) [5], cape aloe (*Aloe ferox*) [6], uzara (*Xysmalobium undulatum*) [7], and to some extent, hoodia (*Hoodia gordonii*) [8], among others [9]. From the 1960s onward, products quickly gained popularity, initially in Germany, then France, and by the mid-1980s, all over the developed world. This led to an increase in demand and consequently harvesting pressure in the countries of origin, to the point that devil’s claw was briefly considered to be listed on CITES appendix II [10]. However, ongoing efforts to introduce good harvesting practices and cultivation attempts helped supply to become more sustainable.

Once harvested, botanical differentiation between species and subspecies is virtually impossible, and it can safely be assumed that since the 1970s, the product of commerce is one or the other and often of mixed origin [11,12,13,14]. Thus, current official compendia do not distinguish between the two botanical sources of devil’s claw but require compliance in terms of contents of the marker compound harpagoside, a cinnamoylated iridoid glucoside. The primary medicinal uses of devil’s claw are the management of arthritis, pain, and dyspepsia [15,16]. An impressive number of clinical trials, the earlier being mostly observational, the more recent randomized, placebo-controlled studies—albeit being of variable quality—indicate clinical efficacy and safety [17]. However, whether harpagoside is more than a just marker, but also the (only) active compound, remains to be demonstrated. Consequently, superiority of *H. procumbens* over *H. zeyheri* cannot be derived merely from harpagoside content [18]. Lower levels of harpagoside do not necessarily translate to lower levels of total iridoids, and phytochemically distinct extracts from *H. procumbens* and *H. zeyheri* have shown similar in vivo analgesic and anti-inflammatory properties [19].

**Figure 1:**
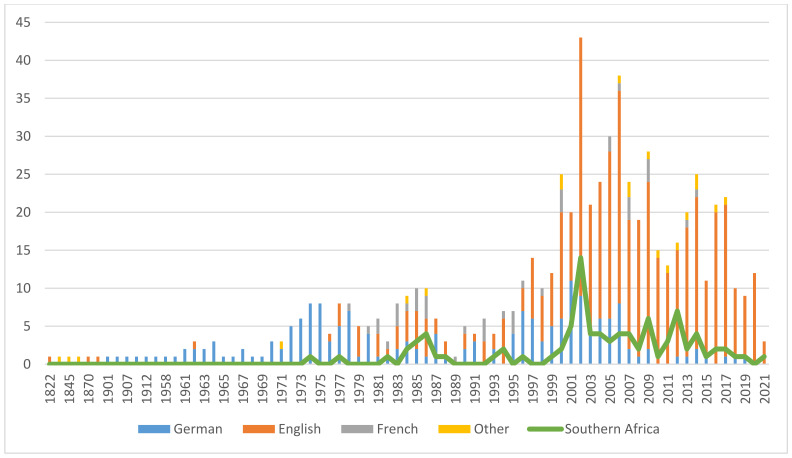
Publications on *Harpagophytum* spp., 1822–2021 (colors indicate publication language/origin of research).

The vast body of evidence presented here—over a period of 55 years, about one general review per year was published in the scientific literature [20,21,22,23,24,25,26,27,28,29,30,31,32,33,34,35,36,37,38,39,40,41,42,43,44,45,46,47,48,49,50,51,52,53,54,55,56,57,58,59,60,61,62,63,64,65,66,67,68,69,70,71,72,73,74,75,76,77,78,79,80], not counting reviews specific to clinical efficacy (see Section 12.1.)—makes devil’s claw one of the best-researched botanicals. Figure 1 illustrates the growing and sustained research interest. The 694 included publications were grouped by language, which yielded a perspective on how research interest spread geographically over time. Despite English becoming the lingua franca of science toward the end of the 20th century, a trend is clearly noticeable—from Germany to France to the rest of the world—and confirmed by research, trade, and availability and popularity of pharmaceutical products. An interesting discrepancy reveals itself when comparing the total with the research output of the region of origin. Nonetheless, knowledge gaps concerning species interchangeability remain to be closed, the elucidation of which is one purpose of this review. It is hoped that the assembly of this extensive bibliography will stimulate further research of this interesting genus of medicinal plants.

## 2. Materials and Methods

Multiple searches were conducted in the PubMed, Scopus, and Google Scholar databases with the following keywords and combinations thereof: “*Harpagophytum*, harpagophyton, devil(’)s claw, Teufelskralle, grapple plant, sengaparile, garra-do-diabo, griffe du diable, (h)arpagoside, taxonomy, nomenclature, ethnobotany, traditional use, ecology, cultivation, sustainability, economy, trade, CITES, chemistry, biochemistry, compounds, pre-clinical, pharmacology, clinical, RCT, safety, toxicology, veterinary, review”. Union catalogues were also searched. The search was limited to scientific literature, and popular magazines and compendia were excluded. Also excluded were articles which only mentioned *Harpagophytum* without elaboration. Further excluded were reports on compounds present in *Harpagophytum*, that were derived from other sources (e.g., harpagoside from *Scrophularia* spp.).

Reference sections of selected publications were searched manually. Academic theses were retrieved primarily via the Bielefeld Academic Search Engine (BASE). Patents were retrieved from the European, US, and international (WIPO) patent office databases.

**Figure 2:**
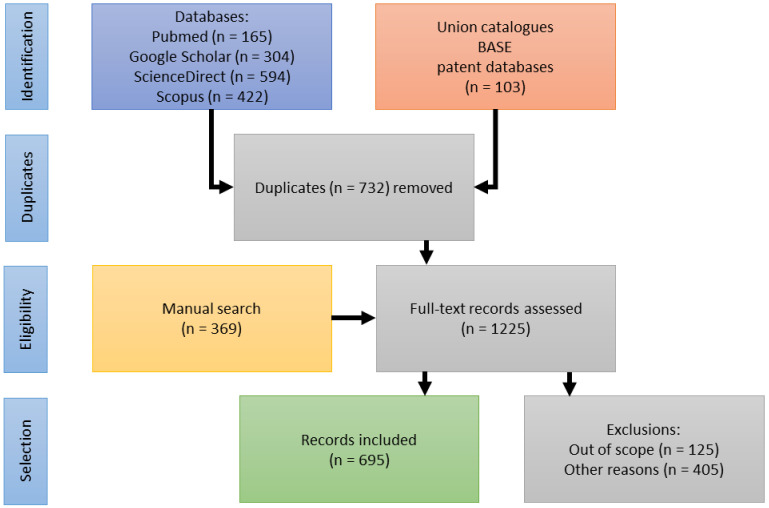
Flow diagram of the reference identification, screening and inclusion.

A substantial body of publications (125) was identified addressing aspects of ecology, stakeholders’ livelihoods, efforts in capacity building, as well as access-benefit-sharing (ABS) and its legislation. They are included in the publication statistics (see Figure 1). In reviewing the pharmaceutical history of devil’s claw, however, these topics appear out of scope and will be reviewed in a separate publication. Figure 2 illustrates the selection process.

## 3. Nomenclature

### 3.1. Taxonomy

The genus *Harpagophytum* was first described as *Uncaria* Burch. by Burchell in his *Travels in the interior of southern Africa* (1822) [81]. However, he was apparently unaware that *Uncaria* had already been used by Schreber for a genus in the Rubiaceae in 1789. Purportedly, de Candolle first noted this oversight, leading Meisner to describe the species as *Harpagophytum procumbens* DC [82]. However, de Candolle’s section of the *Prodomus* was only published in 1845 [83], making Meisner the author of the genus and creating the following complete citation as:

*Harpagophytum* DC. ex Meisner, PI. Vas. Gen. 1: 298 and 2: 206 (1840), syn.: *Uncaria* Burch., Trav. Int. S. Afr. 1:536 (1822), nom. illegit., non Schreb. 1789; type specimen: *Harpagophytum procumbens* (Burch.) DC. ex Meisner, PI. Vas. Gen. 2:206 (1840); basionym: *Uncaria procumbens* Burch., Trav. Int. S. Afr. 1: 536 (1822).

Decaisne, in his review of the Pedalineae, attributed four distinct species to the genus: *H. procumbens* DC., *H. burchellii* Decne. (= *H. procumbens*), and for the first time, *H. zeyheri* and *H. leptocarpum* [*Uncaria leptocarpa* (Decne.) Ihlenf. & Straka] [84]. The genus was last reviewed by Ihlenfeldt and Hartmann (1970) [1], who differentiated two species and five subspecies primarily based on the shape of the fruit correlated with the number of seeds. They also provide the most recent botanical descriptions for all subspecies.

*Harpagophytum procumbens* (Burch.) DC. ex Meisn.:

- H. procumbens (Burch.) DC. ex Meisn. ssp. procumbens—(1).
- H. procumbens (Burch.) DC. ex Meisn. ssp. transvaalense Ihlenf. & H. Hartm.—(2).

*Harpagophytum zeyheri* Decne.:

- H. zeyheri Decne. ssp. zeyheri—(3).
- *H. zeyheri* Decne. ssp. *schijffii* Ihlenf. & H. Hartm.—(4).
- *H. zeyheri* Decne. ssp. *sublobatum* (Engler) Ihlenf. & H. Hartm.—(5).

**Figure 3:**
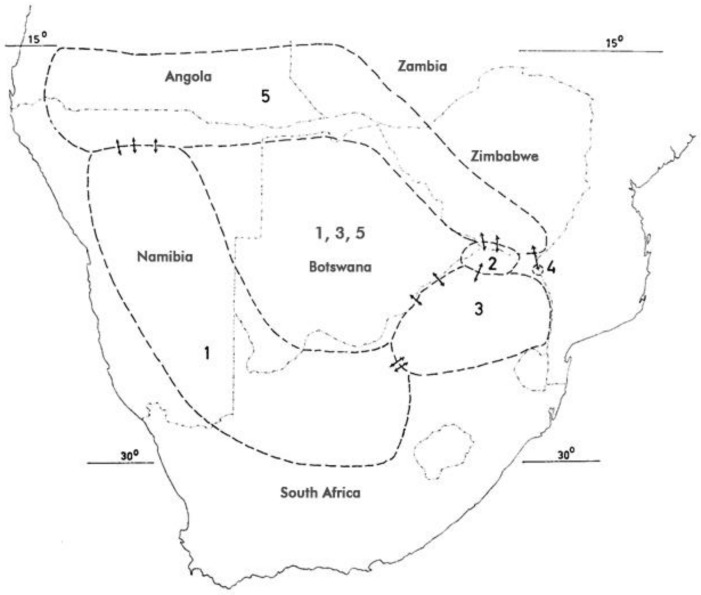
Distribution of H. *procumbens* and H. *zeyheri* (after [1,64,91]). For numerical attribution of species, see Section 3.1. Arrows indicate introgression.

The numbers in parentheses represent the respective species in Figure 3 below.

Synonymy:

- *H. burchellii* Decne. = *H. procumbens* ssp. *procumbens* DC. ex Meisn.
- *H. zeyheri* f. *sublobatum* Engl. = *H. zeyheri* ssp. *sublobatum* (Engl.) Ihlenf. & H. Hartm.
- *H. procumbens* var. *sublobatum* (Engl.) Stapf = *H. zeyheri* ssp. *sublobatum* (Engl.) Ihlenf. & H. Hartm.
- *H. peglerae* Stapf = *H. zeyheri* ssp. *zeyheri* Decne.

Interspecific introgression has been described [85] and shown to be reflected in morphometric measurements, and DNA profiles. Both species and all their putative hybrids also showed geographical variation in biochemical composition [2,85,86,87,88,89,90].

### 3.2. Vernacular Names

Teufelskralle, Trampelkette (Ger.); devil’s claw, grapple plant (Eng.); garra-do-diabo (Port.); garra del diablo (Esp.); artiglio del diavolo (It.); griffe du diable (Fr.); sengaparile (Tswana), duiwelsklou, kloudoring, duiwelsdoring, sanddoring, beesdubbeltjie, wolspinnekop (Afr.); otjihangatene (Oshiherero);//khuripe//khams, gamagu (Nama/Damara); elyata, omalyata (Oshikwanyama); ekatata, makakata (Oshindonga/Kwangali); likakata (Gciriku/Shambyu); !ao!ao,//xsamsa-//oro,//xemta≠’eisa (Kung); ||am-si-||q’oa-ka (West !Xoon), malamatwa (Silozi) [92,93,94].

## 4. Distribution

In the context of species interchangeability in commerce, it is noteworthy that the long-time assumption that only *H. procumbens* occurs in Namibia was disproved as early as the late 19th century. Ihlenfeldt discussed collections from the Etosha pan and later from the Kaokoveld and the Caprivi strip holding specimen of *H. zeyheri* [95]. Baum (1903) reported *H. procumbens* (Burch.) DC. var. *sublobatum* Engl. [= *H. zeyheri* Decne. ssp. *sublobatum* (Engler) Ihlenf. & H. Hartm.] from near lake Camelungo in southern Angola [96]. Cultivation has been experimented with in northern South Africa and, more recently, in Namibia, however, it has thus far neither proven very successful nor commercially viable [97,98,99].

## 5. Ethnobotany

**Figure 4:**
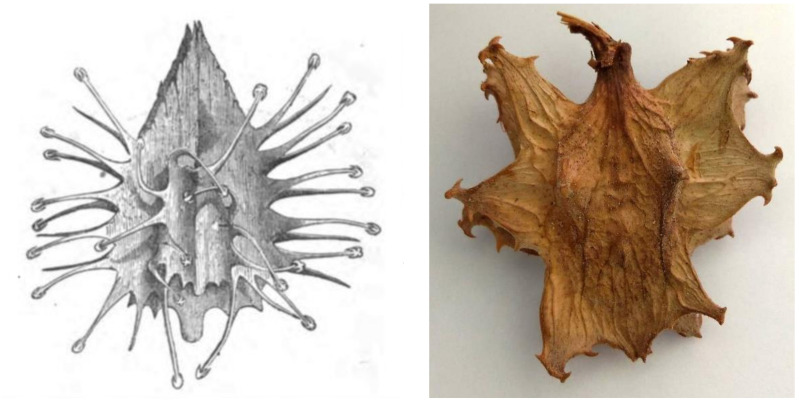
Fruit of the “Grapnel” (note the misspelling!) plant from [101] vs. an actual fruit (photograph by the author).

Interestingly, there are no records for indigenous use of devil’s claw until the beginning of the 20th century. Two accounts from the 19th century by Wood [100] and Cooke [101] (Figure 4) were the only ones that could be found making reference to devil’s claw (as grapple plant—*Uncaria procumbens*) but focus on its “devilish nature”: *“The reader may easily imagine the horrors of a bush which is beset with such weapons. No one who wears clothes has a chance of escape from them. If only one hooked thorn catches but his coat-sleeve, be is a prisoner at once. […] If the reader would like to form an idea of the power of these thorns, he can do so by thrusting his arm into the middle of a thick rose-bush, and mentally multiplying the number of thorns by a hundred, and their size by fifty”* [100].

Lübbert, in 1901, provided the first unambiguous account for the use of “Kuri-Khamiknollen” (= tubers of //khuripe//khams = *Harpagophytum*) in wound healing [102]. In 1907, Hellwig, medical officer of the imperial protection forces in German South-West Africa (Namibia), compiled a report on medicinal plant uses of the indigenous population, including an account of the Herero Samuel Kariko of the use of “otjihangatene” (=*Harpagophytum*) to treat cough, diarrhea, constipation, and venereal diseases [103]. Dinter, in 1909 and 1912 [104,105], utilized this report for his account of local food plants, but unfortunately omitted to include medicinal uses because he considered them unverified [106]. The fact that Hellwig provided an explicit source renders the colorful story of how the use of devil’s claw was “discovered” by Mehnert implausible and more likely part of a marketing strategy (see below) [107].

Later accounts corroborated these early records of traditional use of devil’s claw tubers primarily in the form of infusions and decoctions for digestive purposes, midwifery, pain relief, fever, diabetes, as a general tonic, for infectious diseases, and the dry powder topically as a wound dressing [40,92,108,109,110,111,112]. Ethnoveterinary uses in poultry have also been reported from Botswana [113]. It must be noted, however, that none of the early records clearly differentiate between species. It can only be speculated based on the origin of the records that Nama/Herero may have referred to *H. procumbens,* whereas reports from Botswana would concern mostly *H. zeyheri*.

## 6. Economy

### 6.1. History of Commercialization

The story around how a soldier of the Kaiserliche Schutztruppe (German “imperial protection forces”) and later a farmer in Mariental (Namibia) Gottreich Hubertus Mehnert came across devil’s claw is firmly anchored in the scientific literature. Sometime during the so-called Hottentot uprising from 1904 to 1908 (in fact, a brutal war and genocide of the German troops primarily against the Herero and Nama tribes, which has most recently been recognized by the German government [114]), after observing a local healer successfully improving the condition of a gravely wounded local, he questioned the healer about the magic remedy, but the healer refused to disclose the place from where he had collected it. Purportedly, access to one of the most successful botanical drugs of modern times can be attributed to Mehnert’s pointer dog [107].

**Figure 5:**
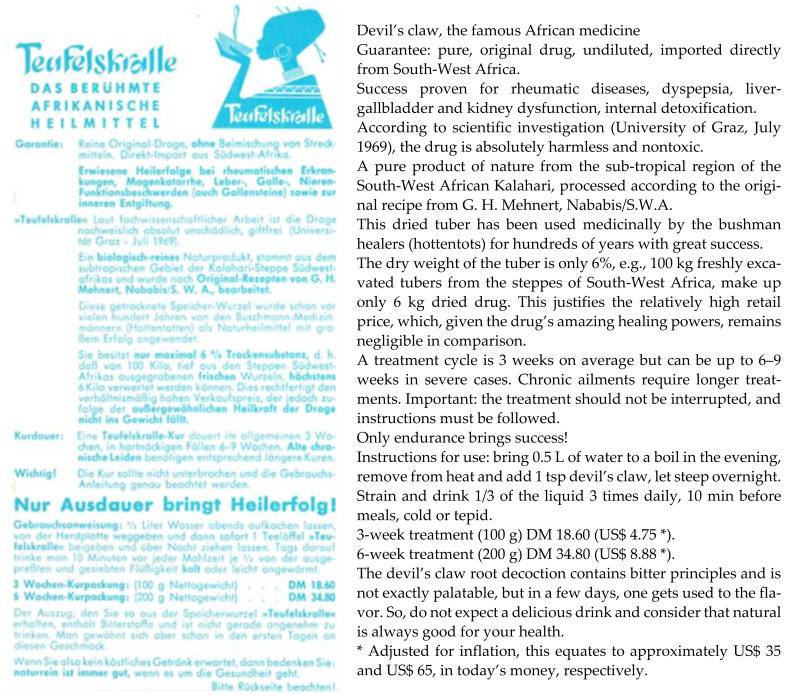
Advertisement Fa. Hagen (early 1970s).

This version, however, must be relegated to the world of “romance” and seen as part of an elaborate marketing campaign—it is repeated in many slightly altered versions by multiple authors. Mehnert doubtlessly experimented with the root and found it effective in a variety of ailments, but the discovery of its medicinal powers ought to be attributed first and foremost to the native tribes and secondarily to Lübbert and Hellwig (see above) with whom they shared their knowledge. It was sheer luck that nobody else developed an interest, allowing Mehnert to consolidate his “research” and to commence commercialization. He eventually shared it, while being interned at camp Andalusia during the 2nd World War, with another “collateral” prisoner, German scientist O. H. Volk, who had visited German South-West Africa at the wrong time [115]. In the camp’s botanical society, knowledge was freely exchanged, which allowed Volk to return home to Germany with likely an entire laundry list of interesting plants. The introduction of devil’s claw (and probably also rooibos) to Germany can be attributed to him [58]. He shared his knowledge with Zorn who conducted some initial pharmacological research [116] and then initiated himself a flurry of investigations elucidating devil’s claw’s basic chemistry [117,118,119,120,121,122,123,124,125,126,127,128]. Meanwhile, in the early 1950s, Mehnert trademarked “Harpago” and started exporting to Germany. Erwin Hagen trademarked “Harpago” in Germany in the early 1960s and began to market it as an infusion and later in homoeopathic preparations [129] (Figure 5). “Harpagosan” tea was registered as a botanical drug in Germany in 1977 [130].

What follows is a story of extensive biochemical, pharmacological, toxicological, and clinical investigation, and the development of multiple standardized pharmaceuticals, initially in Germany (the German drug information system AMIce alone lists a total of 434 products, most of which, however, are no longer active, see, e.g., [131]), and since the 1980s, also in France and elsewhere [132]. Demand quickly started to grow exponentially, and concerns were raised over the sustainability of harvesting practices [133,134,135]. In response to unsustainable harvesting and poor processing practices, the Namibian Devil’s Claw Exporter’s Association Trust became part of a Good Agricultural and Collection Practice (GACP) project in which it intends to ensure that Namibian devil’s claw is sustainably harvested and processed according to GACP guidelines.

### 6.2. Trade

**Figure 6:**
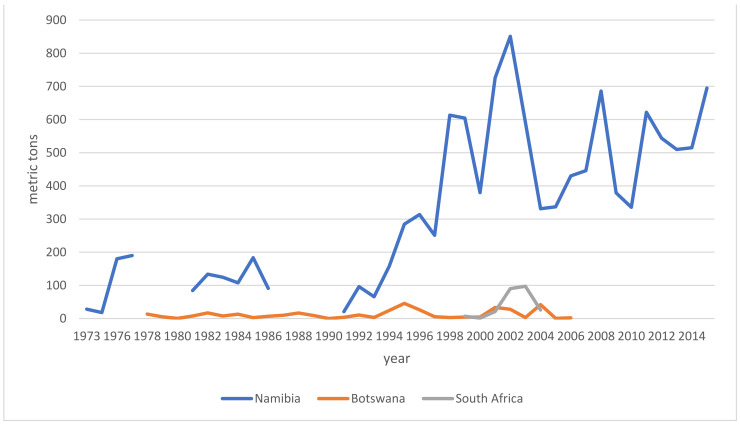
Devil’s claw exports by country—gaps reflect years in which no data was reported.

Market demands impact livelihoods and policymakers alike. Trends indicate the health of an industry and inform resource assessments as well as regulatory interventions. With the following breakdown of trade and export data, I intend to address a controversy around species interchangeability, namely how the ingredient is regulated in the finished product markets. Hagen and others created a demand which local suppliers struggled to meet [133,134,135]. Sustainable collection and harvesting practices and governmental oversight were largely absent until ~1975. When originally only *H. procumbens* had been collected, driven by the economic boom, the collection and admixture of *H. zeyheri* commenced as early as the 1970s [11,12,13,14]. Furthermore, albeit on a much smaller scale than Namibia, both South Africa and Botswana [136,137,138] began to participate in the export market, also adding *H. zeyheri* into the supply chain (for distribution see above). Nott [14] and Taylor and Moss [138] broke down data specific to importing countries and explicitly listed importers, respectively. It is therefore safe to state that all importing markets have received either both species or mixtures thereof as early as the late 1970s. European regulators acknowledged the commercial reality by adding *H. zeyheri* to pharmacopeial monographs (see Section 6), while the US, for instance, remained oblivious to this practice, which stirred a controversy over the regulatory compliance and legitimacy of products containing *H. zeyheri* in 2015 [139]. The following overview of export volumes (Figure 6) is compiled from multiple sources [10,14,18,136,137,138,140,141,142,143,144,145,146,147,148,149,150,151] and further informed by the Namibian Ministry of Environment and Tourism (MET). The MET stopped sharing its data—based on export permits—with the public in 2015. According to one of the most prominent Namibian exporters of devil’s claw, the years 2015–2020 saw a slight increase in demand, peaking in 2019 at around 1000 metric tons, otherwise averaging around 700 metric tons annually. Materials in trade (both species) fall into four categories: conventional (lowest) quality makes up about 80% of the trade volume, GACP quality currently contributes about 10–15% to the total, though efforts are underway to dramatically increase this proportion, certified organic quality adds organic certification to GACP-compliant material and makes up about 5–10% of the total trade volume, and finally, organic and Fair for Life certified material (*H. procumbens* only) contributes ~1% to the trade total. Prices per kg (for full container loads, cost and freight) range from €4.00 (*H. zeyheri*) and €6.70 (*H. procumbens*) for conventional quality, via €5.40 (*H. zeyheri*) and €8.20 (*H. procumbens*) for GACP quality, and €7.20 (*H. zeyheri*) and €8.50 (*H. procumbens*) for organic quality, to €9.00 for Fair for Life certified material (pers. comm. G. Diekmann, EcoSo Dynamics cc, Namibia). While these prices and volumes make this a sizeable industry, it must be noted that most of the value is of course added during the manufacture of pharmaceuticals in the target markets. It is also noteworthy that over all this time, Namibian exports may have been bolstered by (illegal) imports from Angola and Zambia, for which—naturally—no records exist [152].

## 7. Representation in Pharmacopeias and Authoritative Compendia

Given its presence in the European marketplace since the 1950s and in the US at least since the late 1970s, pharmacopeial standards for devil’s claw were set surprisingly late, likely due to suitable analytical methods not being available. While a qualitative assessment for the bitterness value according to the German Pharmacopoeia 7 (DAB 7) was suggested as early as 1977 [11], no specific monograph for devil’s claw was included in DAB until 1993, which, in fact, required testing for harpagoside content (see Table 1 below). The first monograph in Europe appeared in the British Herbal Pharmacopoeia in 1981. Devil’s claw first appeared in the European Pharmacopoeia in 1995, *H. zeyheri*, however, was not included as an allowable source species until 2003. The US Pharmacopeia, on the other hand, does not have a monograph for devil’s claw other than a draft proposal published in the Herbal Medicines Compendium in 2013 [153].

## 8. Biochemistry

After Volk’s return to Germany (see Section 6.1) and following Zorn’s first pharmacological study of devil’s claw in 1958 [116], the university of Würzburg (Germany) became a research hotspot for the elucidation of active and suitable marker compounds in devil’s claw for decades to come. The effort was largely concluded by the end of the 1980s and comparatively little has been added to this effort since. Table 2 lists all publications focused on the biochemical composition. For analytical methods and quality control, see Section 9.

**Figure 7:**
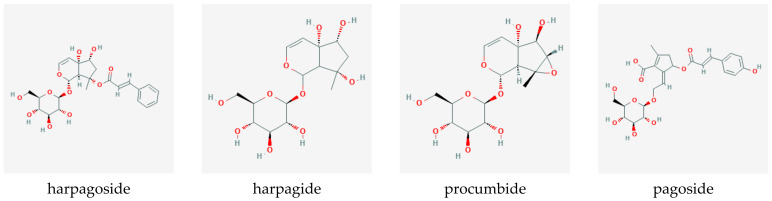
Iridoid glucosides present in devil’s claw root (source PubChem).

Iridoid-glycosides, primarily harpagoside, harpagide, and procumbide; phytosterols; phenylpropanoids such as verbascoside; triterpenes, such as oleanolic acid, 3β-acetyloleanolic acid, and ursolic acid; flavonoids, such as kaempferol and luteolin; unsaturated fatty acids, cinnamomic acid, chlorogenic acid, and stachyose were identified as the most prominent compounds present in the root. Figure 7 shows the chemical structures of the primary iridoid glucosides present in *Harpagophytum* root.

Interestingly, the biosynthetic pathway for harpagoside is not yet well-elucidated. The first step resulting in geranyl diphosphate is still considered to be under debate [17], since while the principal steps are known, some intermediates remain hypothetical and dependent on the “chosen” pathway. Georgiev and colleagues [30] propose two different routes to the formation of geranyl diphosphate from the condensation of dimethylallyl diphosphate and isopentenyl diphosphate, the latter being supplied through either the mevalonate or the mevalonate-independent pathways. Geraniol is synthesized by geraniol diphosphate synthase and hydroxylated to form 8-hydroxygeraniol, followed by two oxidation steps and isomerization into 8-epi-iridodial. Carboxylation and glycosylation form its glycoside, which, in turn, is transformed into harpagide through decarboxylation and oxidations. Finally, harpagoside emerges as the product of cinnamoyl esterification at the 3-hydroxyl position.

Several studies have investigated differences in the quantitative composition of different *Harpagophytum* species, subspecies, and hybrids [19,181,182,183,184], and found the composition to be highly variable, depending on the material used, collection location, natural variation within the taxa, environmental influences, processing, and analytical methods. Content of the marker compound harpagoside is generally lower in *H. zeyheri* and has been found to be between 0%, 1%, and 4% in *H. procumbens* and between 0% and 3% in *H. zeyheri*. Verbascoside and isoverbascoside contents in *H. procumbens* varied between 0.2% and 0.4% and 0.2% and 1%, respectively. Pagoside content in *H. procumbens* varied between 0.06% and 0.16%. Hybrids showed the highest contents for most key compounds except harpagoside. 8-p-Coumaroylharpagide content in *H. zeyheri* varies between 0.7% and 1.4%, while being effectively absent in *H. procumbens*. The lower harpagoside content in *H. zeyheri* has in the past driven controversies over species equivalence in terms of clinical efficacy, however, this debate seems futile as a marker compound is not necessarily the (only) active one. Indeed, the pre-clinical research (outlined in Section 10) indicates that activities of multiple rather than single compounds may contribute to the overall effect.

**Table 2:** Elucidation of the biochemical composition of devil’s claw root.

Topic	Year	Reference
Isolation and characterization of harpagoside	1960	[117]
Stachyose, raffinose, and a further glucoside in the aqueous phase	1961	[118]
Characterization of harpagoside	1961	[119]
Isolation and characterization of harpagoside and harpagide	1962	[120]
Characterization of harpagoside	1962	[121]
Characterization of harpagide	1963	[122]
Isolation of stachyose and a further glucoside	1963	[123]
Characterization of harpagoside	1964	[124]
Isolation of procumbide	1964	[125]
Structural characterization of harpagoside	1966	[126]
Characterization of procumbide and further constituents	1967	[127]
Characterization of procumbide	1968	[128]
Characterization of a chinone and other constituents	1970	[185]
Characterization of procumbide	1971	[186]
Further constituents	1974	[187]
Elucidation of triterpene esters	1975	[188]
Overview of known mono-, di-, and sesquiterpenoids with pharmacological activity	1977	[189]
Elucidation of a resin, an essential oil, and a mucilaginous fraction	1978	[190]
Structural characterization of procumbide	1979	[191]
Glucose, galactose, fructose, myo-inositol, sucrose, raffinose, and stachyose identified	1979	[192]
Preparation and structure of harpagogenine	1981	[193]
Carbohydrates and harpagoside in tissue cultures and roots of devil’s claw	1982	[194]
New iridoids: 8-*O*-(p-coumaryl)-harpagide and procumboside	1983	[195]
Novel iridoid and phenolic compounds	1987	[196]
Three pyridine monoterpene alkaloids from harpagoside and commercial extract	1999	[197]
Review of iridoids	2000	[198]
Review of composition (both species)	2002	[199]
Two diterpenes, (+)-8,11,13-totaratriene-12,13-diol and ferruginol	2002	[200]
New iridoid- and phenylethanoid glycosides	2003	[201]
Acetylated phenolic glycosides	2003	[202]
Pharmacological characterization of harpagoside	2004	[203]
Chinane-type tricyclic diterpenes and other minor compounds	2006	[204,205]
Review of iridoids and other compounds	2006	[206,207]
Review of chemical constituents	2007	[208]
Elucidation and characterization of compounds with specific pharmacologic profiles	2008	[209,210]
New triterpenoid glycoside, harproside, and new iridoid glycoside, pagide	2010	[211]
Kynurenic acid content	2013	[212]
New iridoid diglucoside	2016	[213]

## 9. Analytical Methods and Quality Control

The quickly increasing popularity of devil’s claw products required an ongoing effort to develop and refine tools to identify and quantify devil’s claw in its raw, processed, and finished product states. Initially, the primary aims were identification and contaminants [214,215], later, standardization [11] and quality control [216,217], and finally identification and quantification methods to support pharmacological and clinical research. Early methods, however, did not account for species differentiation, i.e., simple pharmacy-proof methods of the 1970s would likely not have been able to differentiate between *H. procumbens* and *H. zeyheri*. In fact, methods and equipment refined enough to do so, regardless of the extent of processing, only became available in the 1990s. Analysis of retention samples retrospectively determined the presence of both species in commercial products. Table 3 provides a quick reference to publications of methods of quality control in chronological order. In current practice, the most commonly used methods for identification and assaying devil’s claw raw materials and products include TLC, HPLC, HTPLC, and LC/MS, for instance, the current edition of the European Pharmacopoeia employs microscopy and TLC for identification and LC for harpagoside quantification; more recently, chemometric modeling and hyperspectral imaging have emerged as promising methods for species differentiation.

## 10. Processing, Products, Applications

The majority of data on processing and delivery systems is provided in the list of patents compiled in Section 14. EMA’s HMPC assessment report on *H. procumbens* and/or *H. zeyheri*, radix, provides an overview of extracts that are most commonly used in commercial products [167]:

- Liquid extract (1:1; 30% *v/v* ethanol)
- Soft extract (2.5–4.0:1; 70% *v/v* ethanol)
- Dry extract (1.5–2.5:1; water)
- Dry extract (5–10:1; water)
- Dry extract (2.6–4:1; 30% *v/v* ethanol)
- Dry extract (1.5–2.1:1; 40% *v/v* ethanol)
- Dry extract (3–5:1; 60% *v/v* ethanol)
- Dry extract (3–6:1; 80% *v/v* ethanol)
- Dry extract (6–12:1; 90% *v/v* ethanol)
- Tincture (1:5), extraction solvent ethanol 25% (*v/v*)

**Figure 8:**
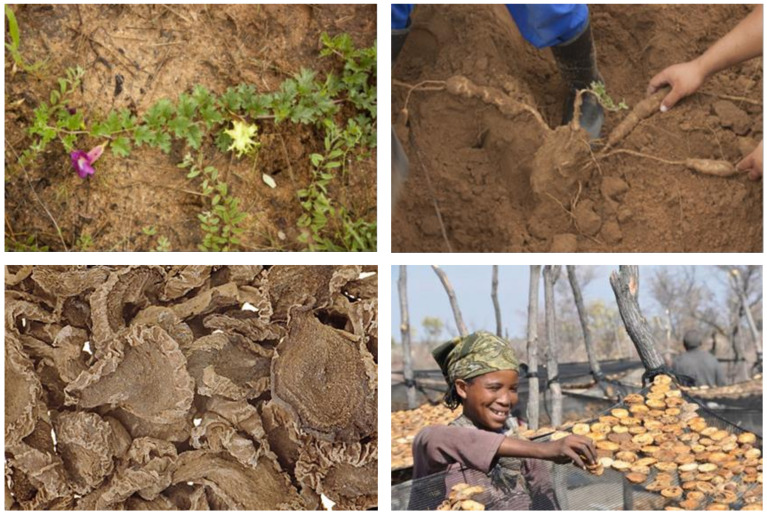
Clockwise: *H. procumbens*, secondary tubers, drying of the sliced tubers, article of commerce (photographs by the author). The article of commerce shown here is conventional quality (see Section 6). Note the difference in color of the slices shown on the bottom right, which were harvested and processed in compliance with GACP.

Figure 8 shows the processing from harvest to the raw material in commerce. Historically, teas [67,274], e.g., Harpagosan (see above), fluidextracts [42,67], spray-dried aqueous extract [26,67], homeopathic preparations for both oral (p.o.) and intraperitoneal (i.p.) application [26,27,67], and powder in capsules [26,67,93] were also common galenic forms. The European Pharmacopoeia stipulates a minimum of 1.2% of harpagoside in the raw material [169]. Dry extracts were standardized to contain a minimum of 1.5% m/m of harpagoside [167].

More recently, Plaizier-Vercammen and Bruwier evaluated the impact of excipients on friability and hygroscopicity of direct compression of a spray-dried *Harpagophytum* extract [275]. Günther et al. analyzed the parameters affecting supercritical fluid extraction with CO_2_ of harpagoside [276]. Performance of a topical preparation with devil’s claw extract on acrylic acid polymers base compared to ketoprofen was assessed by Piechota-Urbanska and colleagues [277]. Both formulations demonstrated rheological stability and high pharmaceutical availability. Almajdoub described a freeze-dried aqueous extract of *H. procumbens* encapsulated in lipid vesicles by using a dry film hydration technique with and without further alginate coating for optimal (delayed) release and small intestine absorption [278]. Development of a gastro-resistant coated tablet prepared from a standardized hydroethanolic root extract for the purpose of more effective delivery and consequent dose reduction was reported by Lopes et al. [279].

## 11. Pre-Clinical Research

### 11.1. Pharmacology

Studies mainly investigated anti-inflammatory activities and were conducted with various extracts, extract fractions, or isolated compounds. *Harpagophytum* iridoid compounds are considered the primary actives, to which anti-inflammatory, antinociceptive, analgesic, antimicrobial, chemopreventive, hepatoprotective, neuroprotective, and immunomodulatory effects are commonly attributed [189,198,209,280,281]. As cyclooxygenase (COX)-1/2 inhibitors have emerged as important targets for treating rheumatoid arthritis, the influence on the arachidonic acid pathway has been a research focus. The most commonly used methods for measuring peripheral analgesic activity were the various forms of the writhing tests, hot-plate test, and the Randall–Selitto test in rats and mice. To demonstrate anti-inflammatory effects, different animal models of inflammation were commonly used, e.g., the carrageenan-induced mouse/rat paw edema, the 12-*O*-tetradecanoylphorbol-13-acetate (TPA)-induced mouse edema, the granuloma pouch test, zymosan-induced arthritis, albumin-induced rat paw edema, adjuvant-induced arthritis in rats (*M. tuberculosis*; Freund adjuvant), and Adriamycin-induced rat paw edema. More advanced in vivo and a variety of in vitro and ex vivo models were developed and employed over time (see Table 4, Table 5, Table 6 and Table 7 below).

Investigated targets for anti-inflammatory effects and their respective IC_50_ (significant inhibitions, primary sources only) are summarized in Table 8.

Table 9 summarizes the results of pre-clinical experiments which studied other effects of *Harpagophytum* and its compounds.

Primary—anti-inflammatory, analgesic/antinociceptive, and antioxidant—effects have been demonstrated in multiple in vitro, in vivo, and ex vivo assays with crude extracts, fractions, and isolated compounds of *Harpagophytum*. However, experiments show some inconsistencies, likely caused by deviations in experimental models and insufficient characterization of the purportedly active compounds, as well as variation in solvent systems [394,395,396]. Further, the consolidated data show that efficacy cannot be clearly attributed to any one of the compounds present in *Harpagophytum*. Focus on harpagoside—albeit serving as a convenient marker—cannot be substantiated in an efficacy context. On the other hand, the presence and effect of verbascoside in *Harpagophytum*, a compound with well-documented anti-inflammatory properties, has not been adequately studied.

### 11.2. Pharmacokinetics

Most of the available pharmacokinetics data were created as a byproduct or in the context of pharmacological experiments with *Harpagophytum* preparations or its compounds. Vanhaelen [52] experimented with harpagoside and harpagide under conditions mimicking those found in the stomach and concluded by suggesting enteric-coated preparations for harpagoside to slow down acid hydrolysis. Chrubasik [217] investigated release and stability of harpagoside in gastric and intestinal fluids and stability for 3 and 6 h, respectively. The author also found harpagoside to be of low bioavailability, a daily dose of 100 mg could not be detected in serum or urine. Chrubasik et al. (2000) [238] established an octanol–water distribution coefficient of approximately 4 that is not dependent on pH or temperature.

Neither *Harpagophytum* ethanolic extract nor harpagoside had a relevant effect on cytochrome P (CYP) 450 3A4 in vitro [397]. An investigation of different *Harpagophytum* extracts elucidated maximum levels of plasma harpagoside after 1.3 to 2.5 h and suggested a correlation between serum harpagoside levels and inhibition of leukotriene biosynthesis in vitro and ex vivo [285,398]. In human liver microsomes and subtype-specific CYP substrates, *Harpagophytum* at a dose derived from [157] activated CYP 2E1 and inhibited CYP 2C19 [399]. Inhibition of CYP 450 was shown for methanolic extracts of H. *procumbens*, and while inhibition of CYP 1A2 and 2D6 was relatively low, moderate inhibition of CYP 2C8/9/19 and 3A4 was noted (IC_50_ between 100 and 350 μg/mL) [400]. However, the impact on drugs metabolized via those enzymes is merely theoretical. Romiti et al. [401] found *Harpagophytum* to interact with the multidrug transporter ABCB1/P-glycoprotein, unrelated to relative harpagoside content. Modarai et al. [402] found *Harpagophytum* preparations, but not harpagoside or harpagide, to weakly inhibit CYP 3A4, but deemed clinical relevance unlikely.

### 11.3. Toxicology

Acute and chronic toxicity have been investigated for the herbal substance, its preparations, and compounds isolated from *Harpagophytum*. Multiple publications cite an unpublished experiment by Albus (1958) in which an LD_50_ in mice was established for a liquid extract (not specified) at 34 mL/kg i.v. and 220 mL/kg p.o. [22,42,51,120]. An LD_50_ in rats was given at 10 g/kg for a spray extract and in mice at 1 g/kg for harpagoside [403]. Vollmann [379] established an LD_50_ of 23 and 10 mL/kg for an infusion and a chloroform/butanolic extract (4:1), respectively. Möse [404], in an unpublished report (cited in [27,44,67,405]) conducted toxicity tests with a *Harpagophytum* infusion in primate and chicken tissue cultures, and no effect on cell development was found, nor did the infusion promote growth of Ehrlich ascites carcinoma in mice. Eichler and Koch referenced toxicity at above 0.5 g/kg without citation [305]. Erdös and colleagues [308] demonstrated *Harpagophytum* aqueous, methanolic, and butanolic extracts to be effectively non-toxic (LD_0_ at 4640 mg/kg p.o. and >1000 mg/kg i.v.), and for harpagoside, a LD_0_ of 395 mg/kg and a LD_50_ of 511 mg/kg. Marzin (1978, cited in [67]) confirmed these results. The same author investigated the toxicity of an extract (2.7% total iridoids), p.o. or i.p., in rats and mice. Administration p.o. was effectively non-toxic, while i.p., some toxicity was observed with a calculated LD_50_ of 10 g/kg (Marzin, 1981, cited in [274]). Vanhalen and colleagues tested toxicity of harpagoside and harpagide in mice and established an LD_50_ of 1 and 3.2 g/kg, respectively [224]. Schmidt [44] elaborated unpublished toxicological investigations with *Harpagophytum* D2 and Harpagosan (DER 2:1) [406,407], establishing an LD_50_ of 20 mL/kg and >30 mg/kg, respectively. Whitehouse et al. [341] established an LD_50_ at 13.5 g/kg p.o. for a *Harpagophytum* root extract (not specified) in mice and no toxicity at 7.5 g/kg over three weeks in rats, while 2 g/kg over one week showed no impact on liver parameters. Ibrahim et al. [408] conducted a battery of toxicity studies in mice (acute, sub-acute, and chronic) with a commercial product (Boiron, France—composition not declared) and found no clinically relevant changes in any of the tested outcomes, attributing a slight increase in liver enzymes to the anti-inflammatory effect. Al-Harbi and colleagues [409] found no oral acute toxicity in mice at 1 and 3 g/kg *Harpagophytum* powder. In a 90-day chronic toxicity study (test substance not characterized), no clinically relevant changes in tested parameters were established, except for a significant decrease in blood sugar and uric acid levels. Both chronic assessments, however, must be considered inadequate due to the insufficiently characterized test material. Allard et al. [410] discussed herb-induced nephrotoxicity, and in that context, called for further investigation of whether a theoretical impact of *Harpagophytum* on major renal transport processes is of clinical relevance. Joshi et al. [411] investigated the toxicology of a *H. procumbens* aqueous-ethanolic extract (1 g/kg/day, equivalent to 7.5–10× the human recommended dose) in male and female Sprague Dawley rats over 4 and 12 weeks. While no significant histopathological effects were found, the study yielded significant—albeit not clinically relevant—sex-related differences in blood chemistry. All these results stand in stark contrast to those of Zorn [116], casting considerable doubt over the authenticity of the plant material used in his experiments.

Mahomed and Ojewole [412,413] conducted experiments in vitro suggesting spasmogenic and uterotonic actions for an *H. procumbens* aqueous extract (10–1000 µg/mL). Whether these results are of clinical relevance in vivo remains to be established (see Section 11.2). Pearson [414] studied the reproductive toxicity of a combination product containing *Harpagophytum* (exact composition not disclosed) for veterinary use in pregnant female Sprague Dawley rats and showed no signs of toxicity. The study, however, is poorly reported and of limited relevance given the unknown composition of the test substance. Contrarily, Davari and colleagues [415] reported teratogenic effects and histopathological changes in fetal tissues (but no significant structural malformations or abnormalities) from an experiment with *H. procumbens* (200, 400, 600 mg/kg) in pregnant Balb/C mice.

## 12. Clinical Research

### 12.1. Efficacy

The efficacy of devil’s claw has been investigated in more than 50 human studies, and case reports and observational studies are summarized in Table 10, while randomized, controlled trials (RCTs) are summarized in Table 11. Indications were primarily degenerative joint diseases as well as low back pain. Trials utilized a variety of methodological designs, with different preparations of devil’s claw and daily doses of harpagoside, varying from <30 to >100 mg. While harpagoside is considered to contribute to the overall activity of devil’s claw preparations, it is not yet fully understood which other compounds may also be of relevance. Furthermore, an investigation into the harpagoside content of commercially available devil’s claw preparations revealed substantial variation, with contents often below the recommended daily dose of 4.5–9 g crude drug (equivalent >50 mg harpagoside) [173,232,233,234,237,416].

Trials have been reviewed systematically with regards to their quality and results concerning safety and efficacy of *Harpagophytum* preparations in publications between 1973 and 2019 [17,23,139,156,417,418,419,420,421,422,423,424,425,426,427,428,429,430,431,432,433]. Another set of reviews considered the efficacy of devil’s claw preparations or its active compounds in specific need states [23,130,434,435,436,437,438,439,440,441,442,443,444,445,446,447,448,449,450,451,452,453,454,455,456,457,458,459,460,461,462,463,464,465,466,467,468,469,470,471,472,473,474,475,476,477,478,479,480,481,482,483,484,485,486,487,488]. All trials observed improvement of the outcome criteria under treatment (some significant), however, significant superiority of the *Harpagophytum* preparations vs. conventional NSAIDs was not reported. This is partly because most trials were observational and/or comparative, while the outcomes of placebo-controlled trials were often inconclusive or overshadowed by methodological deficiencies. Many trials allowed for conventional emergency or co-medication, which further limits the value of the data collected. Despite some studies providing evidence for the effectiveness of certain preparations, the overall quality of evidence is not sufficient. Furthermore, the relevance of early studies with homeopathic dilutions—while included here for completeness’ sake—is limited from a perspective of rational phytotherapy.

**Table 10:** Case reports and observational studies conducted between 1971 and 2021.

Indication	Trial Type, Size	Results	Year	Reference
Chemosis	CR 1	Initial treatment with multiple preparations that did not lead to improvement, then with 300 mg *Harpagophytum* extract (not specified) 3 times daily, orally, for 6 months, leading to drastic improvement.	1983	Belaiche [489]
Familial Mediterranean fever	CR 17	*Harpagophytum* extracts characterized as aqueous (DER 1:2.4, 2.5% harpagoside)—this characterization may also apply to previous trials by Belaiche and Dahout (see above)—6–9 g single dose, duration not provided; significantly decreased recurrence in 80% of patients.	1983	Belaiche [490]
Cancer	CR 2	Tumor regression after taking *Harpagophytum* extract (500 mg daily) and/or Essiac respectively, without cytotoxic therapy.	2009	Wilson [491]
DJD	O ~120	*Harpagophytum* D4–D6, IA, and D1 orally; 1–6 months; substantial improvement of symptoms in most cases.	1971	Beham [492]
CP	O 60	*Harpagophytum* D2, IA, plus tea (2–3 tsp per 1 L water) or 3 × 2 tablets orally, duration not provided; dose-dependent response; 60% substantial improvement of symptoms, 20% improvement, 20% no change.	1972	Schmidt [43]
CP, DJD	O 146	*Harpagophytum* D2, IA, duration not provided; improvement in 134 patients.	1972	Zimmermann, cited in [130]
DJD	O 25	*Harpagophytum* D2–D3, IA, and SC, 1–2 mL, pain-free after 6 injections, or tea (1 tsp per 300 mL) daily for 3–6 weeks.	1972	Brantner [493]
DJD	O 70	*Harpagophytum* D2, IA, some + tea, some + indometacin, duration not provided; improvement in 90% of patients.	1976	Wilhelmer, cited in [44]
CP, DJD	O 21+	*Harpagophytum* D1–D3, IA, SC, and i.v., tea, orally, duration not provided; significant improvement in 30% of patients.	1977	Zimmermann [494]
DJD	O 84	250 or 500 mg *Harpagophytum* extract (not specified) 3 times daily orally for 2–6 months, improvement in 72% of patients.	1979	Dahout, cited in [495]
CP, DJD	O 600	Harpagosan tea (2 tea bags in 500 mL water daily) plus D2 SC for up to 6 months. Symptoms disappeared in 200 patients; 400 patients improved after having received additional conventional medication for the first 3–4 weeks.	1983	Warning cited in Schmidt [44]
Rheumatoid arthritis	O 1	Improvement after treatment with low-potency *Harpagophytum* i.v. and orally, duration not provided.	1987	Stübler [496,497]
DJD	O 553	Patients treated with 2–6 capsules of 400 mg *Harpagophytum* extract (1.5–2.5:1) for 8 to 180 days. Outcomes confirmed RCT results in terms of efficacy and safety.	2000	Müller et al. [498]
DJD	O 255	Post-marketing surveillance study of biopsychosocial determinants and treatment response. Patients treated with *Harpagophytum* extract (60 mg harpagoside/day) for 2 months. Outcome parameters were significantly worse in non-responders.	2009	Thanner et al. [499]
CP, DJD, dyspepsia, hypercholesterolemia, detoxication	O, CR 700+	*Harpagophytum* tea, up to 12 weeks, D2, SC, 20 injections, further improvement with additional D2 i.v. and tea.	1978	Schmidt [130]
Diabetes mellitus with lipometabolic disorder	OT 10	4 patients 3 weeks, 6 patients 4 and 3 weeks, over a total of 6 months; *Harpagophytum* tea, amount not specified; cholesterol, lipid, and blood sugar levels normalized.	1974	Hoppe [500]
Hypercholesterolemia and hyperuricemia	OT 100	*Harpagophytum* tea, 2 tea bags per ½ L water, 3× daily before meals 1/3 of the tea; 20–21 days; lowered cholesterol levels in 80%, normal levels in 45%, 66% improvement in hyperuricemia.	1978	Grünewald [405]
DJD	OT 13	*Harpagophytum* extract (<30 mg harpagoside/day), for 6 weeks, followed up for another six weeks; no overall statistically significant improvements in the conditions.	1981	Grahame and Robinson [501]
DJD	OT 630	42% to 85% of the patients (depending on grouping) showed improvements after 6 months with *Harpagophytum* extract (>90 mg harpagoside/day).	1982	Belaiche [502]
DJD	OT 38	Comparison of *Formica rufa* D6 with *Harpagophytum* D4, for 3 months; improvement in pain severity and mobility with both, *Formica rufa* slightly superior.	1991	Kröner [503]
Effect on eicosanoid biosynthesis	OT 34 (25/8) healthy volunteers	*Harpagophytum*, 4 capsules (500 mg powder, 3% of total glucoiridoids) daily for 21 days. No effect vs. control.	1992	Moussard et al. [504]
MSD	OT 102 (51,51)	Patients treated with *Harpagophytum* extract (30 mg harpagoside/day) or conventional therapy (mainly oral NSAIDs). Number of pain-free patients and changes in Arhus scores after 4 and 6 weeks of treatment was comparable between the groups.	1997	Chrubasik et al. [505]
DJD	OT 43	*Harpagophytum* powder 3 g daily for 60 days. Reduction of pain intensity in 89%, increased mobility in 83%.	1997	Pinget and Lecomte [506]
MSD	OT 2053	Patients treated with *Harpagophytum* extract (30 mg harpagoside/day) for 6 weeks. Symptoms improved over time.	1999	Schwarz et al. [507]
DJD	OT 45	Patients treated with *Harpagophytum* extract (30 mg harpagoside/day) for two weeks plus NSAID treatment, and devil’s claw alone, for four weeks. No worsening of scores was observed during treatment with devil’s claw alone.	2000	Szczepanski et al. [508]
MSD	OT 1026	Patients treated with *Harpagophytum* extract (30 mg harpagoside/day) for 6 weeks. Symptoms improved.	2000	Usbeck [509,510]
MSD	OT 130	Patients treated with *Harpagophytum* extract (~30 mg harpagoside/day) for 8 weeks. Arhus back pain index decreased significantly during treatment. Other measures also improved significantly.	2001	Laudahn et al. [511,512,513]
DJD	OT 583	Patients treated with *Harpagophytum* extract (~30 mg harpagoside/day) for 8 weeks. Symptoms improved and the dose of co-medication (NSAIDs) could be reduced.	2001	Schendel [514]
DJD	OT 675	Patients treated with *Harpagophytum* extract (~30 mg harpagoside/day) for 8 weeks. Efficacy rated good or very good in 82% of cases. The symptom scores decreased, and co-medication was successfully reduced or even discontinued.	2001	Ribbat and Schakau [515]
MSD	OT 250	Patients treated with *Harpagophytum* extract (60 mg harpagoside/day) for 8 weeks. Both generic and disease-specific outcome measures improved.	2002	Chrubasik et al. [516]
DJD	OT 614	Patients treated with *Harpagophytum* extract (480 mg twice daily) for 8 weeks. Symptoms improved in the majority of patients; treatment was well-tolerated.	2003	Kloker and Flammersfeld [517,518]
DJD	OT 75	Patients treated with *Harpagophytum* extract (50 mg harpagoside/day) for 12 weeks. WOMAC index and 10 cm VAS pain scale improved notably.	2003	Wegener and Lüpke [519,520]
MSD	OT 99	Patients treated with *Harpagophytum* extract (~30 mg harpagoside/day) for 6 weeks. Symptoms improved.	2005	Rütten and Kuhn [521]
MSD	OT 102 (29/22/51)	Patients treated with *Harpagophytum* extract (~30 mg harpagoside/day) and/or conventional therapy for 6 weeks. Efficacy was found in all groups, advantages for devil’s claw were not statistically significant.	2005	Schmidt et al. [522,523]
DJD	OT 65	Patients treated with combination of *Harpagophytum procumbens*, *Zingiber officinale,* and *Urtica* sp. (ratio not disclosed) for 8 weeks. Improvements in all efficacy parameters were observed.	2005	Sohail et al. [524]
Endometriosis	OT 6, 12	Patients treated with *Harpagophytum* extract (1600 mg daily) for 12 weeks. Reduction of symptoms in 4 (6) patients after 4 weeks, in all patients after 12 weeks.	2005, 2006	Arndt et al. [525,526]
DJD	OT 259	Patients treated with *Harpagophytum* extract (1.5–3:1, 960 mg daily) and NSAIDs for 8 weeks. At the end of the treatment, 44.8% could decrease NSAID dosage. All parameters improved significantly.	2006	Suter et al. [527,528]
MSD	OT 114	Patients treated with *Harpagophytum* extract (60 mg harpagoside/day) for up to 54 weeks. Most outcome scores improved significantly over time.	2007	Chrubasik et al. [529]
DJD	OT 42	Patients treated with combination of *Harpagophytum* (1800 mg), *Curcuma longa* (1200 mg), and bromelain (900 mg) daily, plus conventional therapies for 2 weeks. Clinically relevant improvement of joint pain scores in all patients.	2014	Conrozier et al. [530]
DJD	OT 20	Patients treated with combination of 500 mg glucosamine sulfate, 400 mg chondroitin sulfate, 10 mg collagen type II, and 40 mg *Harpagophytum* per day for 12 months. Femoral hyaline cartilage thickness significantly improved and radiographic progression of knee osteoarthritis delayed.	2019	Vreju et al. [531]
MSD	OT 39/40/16	Otherwise healthy subjects with mild/moderate neck/shoulder pain related to sport; cream containing a combination of ingredients, including *H. procumbens* root extract + standard treatment, standard treatment, diclofenac patch + standard treatment respectively, for 2 weeks; significant improvement in pain, stiffness, mobility, and working capacity, compared to non-cream groups.	2021	Hu et al. [532]

CP = chronic polyarthritis; IA = intra-articular; SC = subcutaneous; DJD = degenerative joint diseases (osteoarthritis); MSD = musculo-skeletal disorders (low back pain); OT = observational trial; O = observation; CR = case report; NSAID = non-steroidal anti-inflammatory drug; WOMAC = Western Ontario and McMaster Universities.

**Table 11:** RCTs conducted between 1980 and 2017.

DJD	RCT 39	400 mg *Harpagophytum* extract (not specified), and 25 mg diclofenac, or placebo 3× daily for 6 months. Overall confirmation of anti-inflammatory effects without side effects.	~1980	Chaouat, cited in [66,67]
DJD	RCT 50 (25/25)	*Harpagophytum* extract (<30 mg harpagoside/day) and phenybutazone (300 mg per day for the first four days, then 200 mg) respectively, for 28 days. Devil’s claw found equally effective to phenybutazone.	1980	Schrüffler [533]
DJD	RCT 50 (25/25)	Patients treated with *Harpagophytum* extract (<20 mg harpagoside/day) or placebo for three weeks showed a significant decrease in pain severity vs. placebo.	1984	Guyader [534]
DJD	RCT 100 (50/50)	Patients treated with *Harpagophytum* extract (60 mg harpagoside/day) or placebo for 30 days. Only 6 patients in the verum group still experienced moderate pain vs. 32 in the placebo group.	1990	Pinget and Lecomte [535]
DJD	RCT 89 (45/44)	Patients treated with *Harpagophytum* extract (60 mg harpagoside/day) or placebo for two months. Significant decrease in severity of pain and significant increase in spinal and cofexomoral mobility vs. placebo.	1992	Lecomte and Costa [536]
MSD	RCT 118 (59,59)	Patients treated with *Harpagophytum* extract (50 mg harpagoside/day) or placebo for 4 weeks. Treatment group used less analgesics, had greater improvement in median Arhus scores (20% vs. 8%; *p* < 0.059), and had more patients pain-free at the end (9/51 vs. 1/54; *p* = 0.008).	1996	Chrubasik et al. [537,538,539]
MSD	RCT 109 (54/55)	Patients treated with *Harpagophytum* extract (50 mg harpagoside/day) or placebo for 4 weeks. Rescue medication: tramadol. Significant improvement in Arhus index and pain index, and co-medication reduced vs. placebo.	1997	Chrubasik et al. [540]
DJD	RCT 100 (50/50)	Patients treated with *Harpagophytum* extract (30 mg harpagoside/day) or placebo for 30 days. Favorable effects were evident after 10 days vs. placebo.	1997	Schmelz and Hämmerle [541]
MSD	RCT 197 (65/66/66)	Patients treated with *Harpagophytum* extract (50 mg (1), 100 mg (2) harpagoside/day) or placebo (3) for four weeks. 6, 10, and 3 patients were pain-free in groups 1, 2 and 3, respectively. Arhus index score decreased but not statistically significant. Dose-related effect not confirmed.	1999	Chrubasik et al. [542]
DJD	RCT 122 (62/60)	Patients treated with *Harpagophytum* extract (57 mg harpagoside/day) or diacerhein at 100 mg daily for four months. Results showed significant improvement in both groups at a similar rate.	2000	Chantre et al. [543,544]
MSD	RCT 63 (31/32)	Patients treated with *Harpagophytum* extract (~30 mg harpagoside/day) or placebo for 4 weeks. Significant efficacy for visual analogue scale, pressure algometer test, muscle stiffness test, and muscular ischemia test. No differences to placebo in anti-nociceptive muscular reflexes or electromyogram activity.	2000	Göbel et al. [512,513,545,546]
DJD	RCT 46 (24/22)	Patients treated with ibuprofen (800 mg) and *Harpagophytum* extract (~30 mg harpagoside/day) or placebo for 20 weeks. WOMAC scores decreased similarly, but during an ibuprofen-free period, symptoms worsened less than 20% for 71% of devil’s claw patients vs. 41% of placebo patients.	2001	Frerick et al. [547]
DJD	RCT 78 (39/39)	Patients treated with *Harpagophytum* extract (~30 mg harpagoside/day) or placebo for 20 weeks. Co-medication ibuprofen. Symptoms improved similarly for both groups.	2002	Biller [548]
MSD	RCT 88 (44/44)	Patients treated with *Harpagophytum* extract (60 mg harpagoside/day) for 6 weeks or 12.5 mg/day of rofecoxib. Outcome scores improved similarly for both groups. Follow-up confirmed the results of the pilot study.	2003	Chrubasik et al. [538,539,549,550,551,552]
MSD	RCT 97 (36/31/30)	Patients treated with *Harpagophytum* extract (~30 mg harpagoside/day) or NSAID (Voltaren 150 mg or Vioxx 12.5 mg), duration not provided; outcomes show equality of treatment.	2005	Lienert et al. [553,554]
DJD	RCT 60 (30/30)	Patients treated with combination of *Harpagophytum* and *Apium graveolens* extract (cream, 1.5 cm, twice daily) or placebo for 2 weeks. Treatment group showed significant improvement in algometer, flexion, and extension readings.	2006	Pillay [555]
Sore throat after tracheal intubation	RCT 60 (30/30)	Patients treated with *Harpagophytum* extract (480 mg one hour before intubation) or placebo plus premedication (fentanyl, midazolam, propofol). No significant difference was observed between groups.	2016	Anvari et al. [556]
DJD	RCT 92 (46/46)	Patients treated with combination of *Rosa canina, Urtica* sp., *Harpagophytum procumbens*, and vitamin D (20.0 g puree and 4.0 g juice concentrate, 160 mg dry extract, 108 mg dry extract, 5 µg, respectively) or placebo for 12 weeks. WOMAC and quality of life scores significantly improved vs. placebo.	2017	Moré et al. [557]

DJD = degenerative joint diseases (osteoarthritis); MSD = musculo-skeletal disorders (low back pain); RCT = randomized controlled trial; NSAID = non-steroidal anti-inflammatory drug; WOMAC = Western Ontario and McMaster Universities.

### 12.2. Safety

A broad spectrum of claims regarding the safety of *Harpagophytum* in clinical practice can be found in the literature, ranging from unsubstantiated cautioning against its use altogether [215,558] to overly optimistic perspectives in the lay press. The truth, as it often does, lies somewhere in between.

#### 12.2.1. Clinical Safety

Short- and long-term use (on average 30–60 days, in several long-term studies up to 54 weeks) have been described as safe and well-tolerated, and the most reported adverse events in clinical investigations were of mild gastrointestinal nature [559]. These may be related to its anticholinesterase effect in vitro [318,366]. A review of the safety of *Harpagophytum* preparations [560] concluded that they are likely to be safe with only few and no serious adverse events observed, however, it was also established that further, more rigorous safety investigations are required [561], especially considering that the dosage in most studies was found at the lower limit, and for the recommended long-term use.

#### 12.2.2. Interaction Potential

*Harpagophytum* was found to be a weak inhibitor of CYP 1A2 and CYP 2D6, and a moderate inhibitor of CYP 2C8, CYP 2C9, CYP 2C19, and CYP 3A4 in vitro [397,399,400,562], however, clinical relevance is unlikely [402]. Increased anticoagulant effects have been reported with concurrent anticoagulant use [563,564,565,566,567]. While an interaction is possible, evidence is inconclusive [568] and has only been demonstrated in vitro. Herb–drug interactions and interference with anticoagulants are hypothetical and have not been conclusively demonstrated.

#### 12.2.3. Adverse Event Reports

A case of hyponatremia in a patient with systemic hypertension has been associated with *Harpagophytum* (co-medications were losartan, clonidine, omeprazole, and simvastatin) [569]. Another case report suggests development of grade 2 symptomatic hypertension in a normotensive woman during self-administration of *Harpagophytum* [570]. However, available data do not suggest interaction potential with conventional antihypertensives at recommended doses (animal studies demonstrating a hypotensive effect used much higher doses). A case-controlled surveillance study has associated *Harpagophytum* with a pancreatoxic potential [571]. One early case report points at a potential allergic reaction after professional exposure to *Harpagophytum* [572]. Rahman and colleagues [573] included *Harpagophytum* in a review of botanicals with drug-interaction potential in the elderly with inflammatory bowel disease, however, did not present any causality that would justify concern.

#### 12.2.4. Side Effects

Considering the size of the total patient collective from all clinical investigations listed in Section 11.1. (>11,000), and the most common side effects being mild gastrointestinal complaints (nausea, abdominal pain, diarrhea), CNS disorders (dizziness, headache), and allergic skin reactions, the aforementioned case reports should be further investigated, but, until corroborated by new data, their clinical relevance can be deemed as limited.

#### 12.2.5. Pregnancy and Lactation

In Vitro data suggest spasmogenic and uterotonic effects in mammalian uterine muscles [412,413]. In the absence of adequate in vivo data [408,409], use during pregnancy and lactation should be cautioned.

## 13. Veterinary Applications

Veterinary applications of devil’s claw have received increased attention and gained popularity over the last 15 years, with focus on equines and canines. Colas and colleagues [250,255,256,574] provided methods for detection and control of iridoid glucosides from *Harpagophytum* in horse urine. Torfs et al. [575] discussed the potential benefits of devil’s claw products in veterinary practice and cited one study conducted by Montavon [576] in which ten horses with tarsal osteoarthritis were treated with an herbal powder mix containing *Harpagophytum* (20 g total) and smaller quantities of *Ribes nigrum*, *Equisetum arvense,* and *Salix alba* for 10 days a month over three consecutive months. The control group received 2 g of phenylbutazone daily. Locomotor scores improved significantly with the test medication vs. conventional NSAID. However, study results are of limited reliability due to size, lack of blinding, and subjective assessment. Axmann and colleagues [577,578] investigated pharmacokinetics and clinical efficacy of a *Harpagophytum* extract in horses. They provided a method with which they were able to detect harpagoside in plasma for up to 9 h after administration. Efficacy was investigated in a RCT design with 40 horses (20/20), the study medication was 10 g daily of an aqueous *Harpagophytum* extract (25.3% harpagoside) or placebo for 8 weeks, and a follow-up after 16 weeks. Locomotor abnormalities were assessed on a treadmill with an optoelectronic motion capture system, and follow-up was conducted via questionnaires. While the objective motion assessment did not yield significant differences between baseline and the end of the study, evaluation of the questionnaires reflected significant improvements and a “lingering” effect in the subjective assessment.

Moreau and colleagues [579] investigated the efficacy of *Harpagophytum* (harpagoside > 2.7%) as part of a complex mixture of ingredients for improving symptoms of canine osteoarthritis in a RCT with 32 dogs (16 per group) over 8 weeks. The primary endpoint, peak vertical force, was significantly higher in treated dogs vs. placebo after 4 and 8 weeks, and clinical signs overall improved with treatment.

Ethnoveterinary uses of devil’s claw have also been recorded. Moreki [113] reports on ethnoveterinary practices in Botswana to include the use of a decoction of *Harpagophytum* in poultry.

A reliable body of clinical data confirming the efficacy of *Harpagophytum* in veterinary applications is clearly lacking but is needed to better exploit the potential benefits. In this context, it must be noted that the use of devil’s claw—just like other analgesics—is highly restricted in equestrian sport. Harpagoside is included in the “Equine Prohibited Substance List” of the Federation Equestre Internationale as a “controlled medication”, the use of which is prohibited during training and competitions. Curiously, harpagoside is not included in the very same organization’s “List of Detection Times”, leaving horse owners in the dark as to when to discontinue use prior to a tournament. This lack of clarity may further hamper more prolific use in veterinary practice.

## 14. Patents

As mentioned in Section 10, the majority of patents refer to processing methods, specifically extraction and dosage forms, which constitute the only legitimately patentable intellectual property for the pharmaceutical industry, except in cases where new effects or combinations, not previously described in ethnobotanical use accounts, were elucidated. It is noteworthy that most of the earlier patents listed below in Table 12 (pre-2000) have expired or been withdrawn. Pending patents have been excluded.

## 15. Discussion and Conclusions

Devil’s claw is a well-established phytopharmaceutical. A large body of data exists in which composition, pharmacological activities, and clinical effects are elucidated, and in turn support and affirm traditional use applications. Nonetheless, several aspects requiring further investigation were highlighted by this review.

Revision of the genus to account for introgression, geographical, and biochemical variation, and geo-authenticity is needed.

In view of the interchangeable use of both *Harpagophytum* species and mixtures thereof in clinical practice, further comparative examination of the composition of both species is needed. Verbascoside as an anti-inflammatory compound present in *Harpagophytum* could be an interesting target of future research.

Despite some inconsistent outcomes and contradictory results, pharmacological evidence appears to be overall sufficient to support clinical use. Sufficient pharmacological differentiation between *Harpagophytum* species, however, is lacking.

Toxicological evaluations of *Harpagophytum* indicate a low toxicity in animal models. While genotoxicity testing is part of the regulatory requirements for the market authorization of herbal medicinal products in Europe, results are proprietary (product-related) and have not been published. Adequate tests on reproductive toxicity, genotoxicity, and carcinogenicity, performed according to currently valid OECD guidelines, need to be made publicly available.

While there may be strong clinical evidence that devil’s claw preparations are effective in the treatment of degenerative joint diseases and musculoskeletal disorders in principle, this conclusion cannot be extended to specific preparations, because of the varying pharmaceutical quality of individual preparations.

Further investigations are required (a) to identify the therapeutically active substances or fractions and thus enable tests which (b) use accordingly standardized and sufficiently dosed preparations with a carefully designed setup and methodology in order to obtain quantifiable results for the efficacy of devil’s claw preparations. These need to be conducted with both *Harpagophytum* spp. individually but prepared identically. Trial designs should be guided by the recommendations of the International Council for Harmonization of Technical Requirements for Pharmaceuticals for Human Use (ICH). Specifically, both species could be compared in a two-arm cross-over design. Conventional medication could be added as a third arm to assess comparative efficacy. Studies should be of adequate power, randomized, placebo-controlled, and double-blinded. Problematic in an ethical sense is the denial of “first aid” medication in placebo-controlled studies, permission of which would confound outcomes. Outcomes should be objective or at least a combination of objective and subjective measures.

Further research is also warranted in the area of clinical safety, specifically with regard to the drug interaction potential of devil’s claw preparations. Until then, safety considerations as expressed in current compendia, e.g., [15], should be considered appropriate.

## Figures and Tables

**Table 1:** Representation of devil’s claw in pharmacopeias and authoritative compendia.

Source	Species Included	Year	Reference
**Official monographs**			
British Herbal Pharmacopoeia	*H. procumbens*	1981	[154]
Pharmacopée française	*H. procumbens*	1989	[155]
Kommission E	*H. procumbens* (corrected) Monograph was informed by [65,156]	1990	[157]
Pharmacopée française	*H. procumbens* dry extract	1992	[158]
DAB 10 2nd. Supplement	*H. procumbens*	1993	[159]
European Pharmacopoeia 3rd ed.	*H. procumbens*	1997	[160]
European Pharmacopoeia 4th ed. Suppl. 4.3	*H. procumbens/H. zeyheri* (revised)	2003	[161]
Pharmacopée française	*H. procumbens/H. zeyheri* (homoeopathic preparations)	2007	[162]
European Pharmacopoeia 7th ed.	Devil’s claw dry extract	2008	[163]
Health Canada	*H. procumbens*	2008	[164]
European Pharmacopoeia 7th ed.	*H. procumbens/H. zeyheri* (revised)	2011	[165]
Polish Pharmacopoeia 8	*H. procumbens/H. zeyheri*	2008	[166]
USP Herbal Medicines Compendium	*H. procumbens/H. zeyheri* (draft)	2013	[153]
European Medicines Agency (EMA)	*H. procumbens/H. zeyheri* (revised from 2008)	2016	[15,167,168]
European Pharmacopoeia 9.6	*H. procumbens/H. zeyheri* (revised)	2018	[169]
State Pharmacopoeia of Ukraine	*H. procumbens/H. zeyheri*	2018	[170]
Health Canada	*H. procumbens/H. zeyheri* (revised from 2008)	2018	[164]
**Authoritative compendia**			
ESCOP	*H. procumbens*	1996	[171]
ESCOP	*H. procumbens* (revised) (omission of *H. zeyheri* is discussed in [172,173])	2003	[16]
World Health Organization	*H. procumbens*	2007	[174]
African Herbal Pharmacopoeia	*H. procumbens*	2010	[175]
Martindale	*H. procumbens* (continuously revised from 1997)	2017	[176]
**Other compendia**			
Longwood Herbal Task Force	*H. procumbens/H. zeyheri*	1999	[177]
Herbal Medicines	*H. procumbens/H. zeyheri*	2015	[178]
Phytopharmacy	*H. procumbens/H. zeyheri*	2015	[179]
Kooperation Phytopharmaka	*H. procumbens*	2020	[180]

**Table 3:** Analytical methods and methods of quality control.

Topic	Year	Reference
Macroscopic and microscopic descriptions	1964	[58]
Macroscopic, microscopic, and chromatographic differentiation of commercial drug samples	1973	[218]
Macroscopic, microscopic, and chromatographic differentiation of commercial drug samples	1974	[219]
Simple TLC with *Scrophularia nodosa* as a reference standard	1975	[220]
Distribution of harpagoside within *H. procumbens* and *H. zeyheri*	1977	[221]
Standardization by determination of harpagoside, bitterness value, and dry residue	1977	[11]
Spectrometric method for the quantitative evaluation of the glycoiridoids	1978	[222]
Report of falsified, adulterated, and contaminated commercial products	1978	[214]
Quantitative determination of harpagoside via HPLC	1980	[223]
GLC method for the determination of harpagide and harpagoside	1981	[224]
Histological characteristics under scanning electron microscope	1984	[225]
Stability of iridoids during extraction	1985	[226]
Determination of harpagoside, luteolin, chlorogenic, caffeic, and cinnamic acid from extracts	1986	[227]
Analysis of permethylated iridoid glycosides by GC/MS	1986	[228]
Determination of harpagide, 8-p-coumaroyl harpagide (8-PCHG), and harpagoside by HPLC	1994	[229]
Analysis of the harpagoside content of commercial samples by HPLC	1995	[230]
TLC method for determination of harpagoside	1995	[231]
HPLC/UV for the determination of harpagoside in commercial powdered dry extracts	1996	[232]
HPLC/UV for the determination of harpagoside in commercial tea products	1996	[233]
HPLC/UV for the determination of harpagoside in commercial products (multiple dosage forms)	1996	[234]
HPTLC for quantitative determination of harpagoside	1996	[235]
HPLC determination of harpagide, 8-PCHG, and harpagoside in *H. procumbens* and *H. zeyheri*—ratio of harpagoside/8-PCHG can be used to distinguish species	1997	[19]
HPLC determination of ratio of harpagoside/8-PCHG, 8-PCHG < 8% proposed for *H. procumbens*	1998	[12]
Methods for quality control and stability testing of *Harpagophytum* homeopathic preparations	1998	[236]
HPLC/UV for the determination of harpagoside in commercial dry extract products	1999	[237]
Differentiation of *H. procumbens* (<9% 8-PCHG), mixtures (10–30% 8-PCHG), and *H. zeyheri* (>31 8-PCHG) proposed	2000	[13]
Biopharmaceutical quality, release of active ingredients in vitro, and disintegration tests	2000	[238]
Methods for detection of adulterations and contaminations	2001	[239]
Bioequivalence of *Harpagophytum* products	2002	[240]
Near infrared spectroscopy (NIRS) determination of harpagoside, 8-PCHG, and their ratio	2003	[241]
NIR-FT-Raman spectroscopy for identification and quantification of harpagoside	2005	[242]
Determination of harpagoside from CO_2_-extracts with HPLC and HPTLC-densitometry	2005	[243]
NIRS determination of harpagoside, 8-PCHG, and their ratio	2005	[244]
Fast HPLC determination of harpagoside using a monolithic silica column	2005	[245]
Validation of a fast-HPLC for separation of iridoid glycosides to distinguish between species	2005	[246]
LC-DAD-MS/SPE-NMR hyphenation for identification of isobaric iridoid glycoside regioisomers	2005	[247]
X-ray fluorescence spectrometry (SRTXRF) to determine trace elements	2005	[248]
Determination of aflatoxin B_1_	2006	[249]
LC/MS determination of harpagoside, 8-PCHG, and their ratio	2006	[250]
Computational study to estimate the proton and sodium cation affinities of harpagide	2006	[251]
Quality parameters of finished products in the German market	2006	[252]
Proposal to revise the drug–extract ratio of aqueous/ethanolic extracts	2006	[253]
Methods for determination of minerals and heavy metals	2007	[254]
Analysis of iridoids in horse urine	2008	[255]
Solid-phase extraction for LC/MS analysis of harpagoside, 8-PCHG, and harpagide in equine plasma	2008	[256]
Validated HPTLC method for the determination of harpagoside	2008	[257]
High-Pressure Liquid Chromatography-Diode Array Detection (HPLC-DAD) for harpagoside and isoacteoside contents	2009	[258]
HPLC-DAD and HPLC–ESI-MS analyses of stability of the constituents	2011	[181]
Anatomical study of secondary tubers and quantification of harpagoside by HPLC	2012	[259]
Authenticity and contamination tests by DNA barcoding	2013	[260]
Exploring species substitution through chemometric modeling of ^1^H-NMR and UHPLC-MS	2014	[182]
Mid-infrared spectroscopy and short-wave infrared hyperspectral imaging for qualitative assessment of *H. procumbens* and *H. zeyheri*	2014	[85,88]
Morphology, histochemistry, and ultrastructure of foliar mucilage-producing trichomes	2014	[261]
NMR-based chemometric approach for species differentiation	2014	[262]
UPLC Q-TOF ESI determination of harpagosides in *H. procumbens*, *H. zeyheri,* and extracts	2016	[263]
Loss on drying and total ash	2016	[264]
Comparison of microwave and ultrasound-assisted with conventional solvent extraction methods for harpagoside determination	2016	[265]
Innovative micro-extraction techniques to determine harpagoside and phenolic patterns in H. *procumbens* and finished products	2017	[266]
Determination of suitable extraction solvent	2017	[267]
GC-MS determination of chemical constituents	2017	[268]
DNA barcoding to detect contamination and substitution	2017	[269]
HPLC and MS analyses of spagyric tinctures	2019	[270]
Validated RP-HPLC-PDA method for quantification of harpagoside in extracts and finished products	2019	[271]
UPLC–MS profiling of samples from different locations	2019	[90]
Determination of macro- and micro-elements in finished products using ICP OES	2020	[272]
HPLC method for harpagoside determination in finished product (tablet)	2020	[273]

**Table 4:** In Vitro experiments regarding analgesic/antinociceptive and anti-inflammatory effects of devil’s claw preparations and compounds.

Study	Year	Reference
Guineapig isolated ileum; harpagoside (40 µg/mL) and harpagogenine (2.5 µg/mL) non-selectively inhibited contractions; harpagide (40 µg/mL) increased the cholinergic response without inhibitory effects.	1981	Fontaine et al. [282]
Calcium ionophore-stimulated mouse peritoneal macrophages; harpagoside and harpagide inhibited leukotriene C4 (LTC4) and prostaglandin E2 (PGE2) release (not significant) and harpagoside inhibited thromboxane B2 (TXB2) release, similar to ibuprofen.	2000	Benito et al. [283]
Lipopolysaccharide-stimulated primary human monocytes; Harpagophytum * extract, harpagoside, and harpagide extract prevented synthesis of tumor necrosis factor alpha (TNF-α), isolated substances showed no effect.	2001	Fiebich et al. [284]
Ionophore A23187 stimulated Cys-LT levels in anticoagulated whole blood; *Harpagophytum* extract, harpagoside, and extract fractions; inhibitory effect stronger with extract than harpagoside, no effect with fractions without harpagoside, suggesting relation between serum harpagoside and inhibition of leukotriene biosynthesis.	2001	Loew et al. [285]
Modified Hens-Egg-Test at the Chorion-Allantoin-Membrane (HET-CAM) and lipoxygenase assay; ethanolic extracts of *Harpagophytum* (60%, 30%, 0%); 30% most potent in HET-CAM, 60% most potent in inhibiting lipoxygenase pathway.	2002	Wahrendorf et al. [286]
Human neutrophile elastase (HNE); *Harpagophytum* extract, fractions, and isolates; weak dose-dependent inhibition was observed, with *H. procumbens* extract twice as strong as *H. zeyheri*; 6′-*O*-acetyl-acteoside (not in *H. procumbens*) the strongest isolate, followed by isoacteoside and pagoside (dominant in *H. zeyheri*).	2002, 2003	Boje [199]; Boje et al. [201]
Lipopolysaccharide (LPS)-induced inflammation in mouse fibroblast cell line L929; 3-(4,5-dimethylthiazol-2-yl)-2,5-diphenyltetrazolium bromide (MTT) assay, reverse transcription-polymerase chain reaction, PGE2 immunoassay, and nitric oxide (NO) detection; aqueous *H. procumbens* extract; suppression of PGE2 synthesis and NO production.	2003	Jang et al. [287]
Human chondrocytes stimulated with interleukin (IL)-1β; *Harpagophytum* dry extract (210, 480 mg); immunofluorescence and Western blot analyses showed dose-dependent suppression of matrix metalloproteinases production via inhibition of cytokine expression.	2004	Schulze-Tanzil et al. [288]
Bovine and human chondrocytes, stimulated with LPS and IL-1β, respectively; *Harpagophytum* extracts (100, 33, 1 µg/mL); significant suppression of PGE2 expression and NO synthase in human chondrocytes (bovine experiment was flawed).	2006	Chrubasik [289], Chrubasik et al. [290], Hadzhiyski et al. [291]
Human whole-blood assay, human polymorph nuclear leucocytes (PMNL) assay; COX-2, 5-lipogenase (LOX) inhibition, respectively; comparison of *Harpagophytum* aqueous-ethanolic and CO_2_ extracts (2%, 20%, and 30% harpagoside, respectively); variable but weak PGE2 inhibition for all, superiority of CO_2_ extracts in 5-LOX inhibition.	2006	Günther et al. [292]
Human HepG2 hepatocarcinoma and RAW 264.7 macrophage cell lines; harpagoside (0.1–200 µM); LPS-induced mRNA, COX-2 expression, and inducible nitric oxide (iNOS) inhibited, and NF-κB activation suppressed.	2006	Huang et al. [293]
LPS-stimulated THP-1 cells; incubated with 50 µg/mL *H. procumbens* dry extract (DER 1.5–3); microarray (gene chip) assay; noted inhibition of several inflammatory targets.	2009	Balthazar et al. [294]
COX-2 (ovine) enzyme, stimulated by arachidonic acid and TMPD; *H. procumbens* extract, harpagoside, and harpagide; direct inhibition (68%) of COX-2, harpagoside, and harpagide contributed 1.5% and 13%, respectively.	2011	Ebrahim and Uebel [295]
Isolated murine macrophages; *H. procumbens* crude methanolic extract, harpagoside, phenylethanoid-containing fraction, verbascoside; strong inhibitory action related to NO and TNF-α and IL-6 production, and COX-1 and COX-2 expression, comparable to harpagoside.	2011	Gyurkovska et al. [296]
LPS-stimulated human monocytes and mouse RAW264.7 macrophages; molecular targets; *H. procumbens* ethanolic extract (2.9% harpagoside); dose-dependent inhibition of TNF-α, IL-6, IL-1β, PGE2, and COX-2, inhibition of activator protein (AP)-1 pathway without affecting NF-κB and mitogen-activated protein (MAP) kinase pathways.	2012	Fiebich et al. [297]
Pre-transdermal and post-transdermal COX-2 inhibition and permeation studies; *H. procumbens* extract, harpagoside, harpagide; hydroxypropyl cellulose gels (carrier) with permeation enhancers tested on synthetic membranes, with and without enhancers on human skin, Azone^®^ enhancer chosen, direct COX-2 inhibition maintained (pre-permeation 80%, post-permeation 77% COX-2)	2013	Ebrahim [298]
LPS-stimulated monocytic THP-1 cells; enzyme-linked immunosorbent assays, WST-1 assay; *Harpagophytum* extract; dose-dependent suppression of TNF-α, IL-6, IL-8, independent from external metabolic activation.	2014	Hostanka et al. [299]
Primary human osteoarthritis chondrocytes; harpagoside (600 µM); significant reduction in IL-1β-induced expression of IL-6, no effect on nuclear levels of NF-κB.	2015	Haseeb et al. [300,301]
Differentiated 3T3-L1 adipocytes; harpagoside; activation of peroxisome proliferator-activated receptor (PPAR)-γ, significant inhibition of TNF-α-induced mRNA synthesis and production of atherogenic adipokines including IL-6, plasminogen activator inhibitor-1, and monocyte chemoattractant protein-1.	2015	Kim et al. [302]
IFN-γ/LPS-stimulated THP-1 cells; harpagoside and harpagide; decreased TNF-α-secretion in PMA-differentiated THP-1 cells, positive effect on TNF-α and intercellular adhesion molecule-1 mRNA-expression in undifferentiated cells.	2016	Schopohl et al. [303]
Human synovial membranes from subjects with and without osteoarthritis; *H. procumbens* extract, multiple solvents; cannabinoid type 2 (CB2) receptor enhanced, phosphatidylinositol-specific phospholipase C β2 downregulated with water and DMSO, fatty acid amide hydrolase (FAAH) activity inhibited with all.	2020	Mariano et al. [304]

* Species not specified; however, all specific attribution must be cautioned against due to the frequent admixture.

**Table 5:** In Vivo experiments regarding analgesic/antinociceptive and anti-inflammatory effects of devil’s claw preparations and compounds.

Study	Year	Reference
Formaldehyde-induced arthritis in rats; Harpagophytum * infusion p.o. and subcutaneous; significant reduction of swelling, subcutaneous application better tolerated.	1958	Zorn [116]
Albumin-induced paw edema, granuloma-pouch-test, formaldehyde-induced arthritis in rats, rabbit ear-withdrawal test; whole extract and harpagoside, intravenous (i.v.) and i.p.; some (significant) effects shown similar to those of phenylbutazone.	1970	Eichler and Koch [305]
Rats; blood panel; *Harpagophytum* aqueous extract 3:1, 30 mg/kg; triglycerides, uric acid, urea, and cholesterol significantly reduced.	1974	Int. Bio Research [306]
Dextran-induced paw edema; rats; *Harpagophytum*, aqueous extract 3:1; edema significantly reduced.	1974	Int. Bio Research [307]
Eight *Harpagophytum* dry extracts, p.o. and i.v., tested for analgesic and antiphlogistic effects in five animal models; some analgesic and antiphlogistic effects with methanolic, butanolic, and fluid extracts; pure harpagoside superior, semi-chronic models showed better results.	1978	Erdös et al. [308]
Carrageenan-induced rat paw edema (30) and adjuvant-induced arthritis in rats (40); *Harpagophytum* 100–1000 mg/kg, single dose and 21 days; no significant effect in the edema model, some effect in the arthritis model at the higher dose.	1979	McLeod et al. [309]
Carrageenan-induced rat paw edema; aqueous ethanolic crude extract of *Harpagophytum* and various fractions; only crude extract effective, concludes that harpagoside is likely not the (only) active.	1986	Duband [274]
Carrageenan-induced rat paw edema; methanolic extract of *Harpagophytum*; dose-dependent edema inhibition.	1990	Mánez et al. [310]
Carrageenan-induced rat paw edema; aqueous extract of *Harpagophytum* (1.8% harpagoside) and harpagoside i.p.; significant reduction of edema with extract, not with harpagoside.	1992	Lanhers et al. [311]
Adriamycin-induced rat paw edema; *Harpagophytum*, 37, 370, and 3700 mg/kg; dose-dependent edema inhibition up to 48% after one hour; compared to control (Adriamycin only) effect transient after 5 days.	1992	Jadot and Lecomte [312]
Carrageenan-induced mouse paw edema and TPA-induced mouse ear edema; harpagoside (p.o. and topically); no notable protective effects.	1994	Del Carmen Recio et al. [313]
Carrageenan-induced rat paw edema; aqueous extracts of *Harpagophytum* (400 and 800 mg/kg, 2.72% harpagoside) i.p. pre-treatment, p.o., and intraduodenally; significant inhibition i.p. and intraduodenally, no effect orally.	1994	Soulimani et al. [314]
Carrageenan-induced mouse paw edema; *Harpagophytum* and *Uncaria tomentosa* extracts; no effect on inflammatory response individually, but significant effect combined.	2002	Abe et al. [315]
Freund’s adjuvant-induced arthritis in rats; acute (25, 50, or 100 mg/kg) or chronic (100 mg/kg) treatments with *H. procumbens* solution; increased ‘latency of paws’ withdrawal and reduction in paw edema, compared to control.	2004	Andersen et al. [316]
Fresh egg albumin-induced pedal edema in rats, hot-plate and acetic acid tests in mice; *H. procumbens* root aqueous extract (50–800 mg/kg i.p.); significant effect against nociceptive pain stimuli and significant, dose-dependent reduction of edema.	2004	Mahomed and Ojewole [317], Mahomed [318]
Carrageenan-induced back-paw edema, Freund’s adjuvant-induced arthritis, cotton pellet-induced granuloma, and writhing tests in rats and mice; *Harpagophytum* aqueous extract (800 mg/kg bw), acetyl salicylic acid and indomethacin as controls; significant effects in all models similar to indomethacin and acetyl salicylic acid.	2005	Ahmed et al. [319]
TPA-induced COX-2 expression in mouse skin; *Harpagophytum* methanolic extract (200, 400 µg) topically prior to TPA application; significant inhibition of COX-2 expression, COX-1 unchanged, no effect on NF-κB.	2005	Kundu et al. [320]
Carrageenan-induced back-paw edema in rats; *H. procumbens* extract (100, 200, 400, or 800 mg/kg) p.o. and i.p.; reduced intensity of inflammatory response when given i.p.	2006	Catelan et al. [321]
Adult female white New Zealand rabbits, anterior cruciate ligament transected, and medial meniscus removed; *Harpagophytum* extract (150 mg/day), standard food pellets as control; outcome suggests suppression of metalloproteinase-2 production.	2006	Chrubasik et al. [322], Chrubasik [289]
Male ICR mice; formalin test; *Harpagophytum* extract (1.9% harpagoside, 30–300 mg/kg); significant dose-dependent attenuation of licking/biting and spinal nitrites/nitrates.	2008	Uchida et al. [323]
Rabbits after unilateral meniscectomy and transection of the anterior cruciate ligament; thickness, surface area, and volume of the tibial condylar cartilage per MRI; *H. procumbens* extract (14% harpagoside); difference in thickness and volume between healthy and operated leg slightly but not significantly smaller with *Harpagophytum*.	2011, 2014	Wachsmuth et al. [324], Wrubel [325]
BALB/c mice infected with *Salmonella enteritidis*; leukocytes, neutrophils, and mononuclear cell counts, TNF-α, IL-4, 10, 12, histopathological analysis of the liver and small intestine; *H. procumbens* extract (150 µg/day); downregulation of cell counts, TNF-α, IL-10 m 12, IL-4 increased, histopathology of liver unchanged, hypertrophy in the small intestine, reduced with *Harpagophytum*.	2014	Bisinotto [326]
Male SD rats; plantar incision and spared nerve injury; mechanical withdrawal threshold (MWT) test and ultrasonic vocalization (USVs); *H. procumbens* ethanolic extract (300 mg/kg, p.o.); MWT significantly increased, USVs reduced.	2014	Lim et al. [327]
Rats; carrageenan-induced mechanical allodynia and thermal hyperalgesia, involvement of the hemeoxygenase (HO)-1/carbon monoxide (CO) pathway; *H. procumbens* extract (300 and 800 mg/kg i.p.); pretreatment with HO inhibiter reduced anti-hyperalgesic effect, pretreatment with hemin- or CO-releasing molecule induced antiallodynic response.	2015	Parenti et al. [328]
Rats; formalin-induced damage to cartilage tissue; combination of glucosamine hydrochloride, chondroitin sulfate, methylsulfonylmethane, *Harpagophytum* extract (3% harpagoside), and bromelain extract (500 mg/kg); malondialdehyde, NO, 8-hydroxyguanine, IL-1β, and TNF-α significantly lowered, glutathione significantly increased.	2015	Ucuncu et al. [329]
Rats, chronic constriction injury (CCI) of left sciatic nerve model; *Harpagophytum* extract + morphine, each at sub-analgesic dose; significant antiallodynic and anti-hyperalgesic effect suggesting synergistic effect.	2016	Parenti et al. [330]
Immunological angiogenesis induced by bronchoalveolar lavage (BAL) cells grafted into BALB/c mice skin; ethanolic extract of *Harpagophytum*, *Filipendula ulmaria*, and *Echinacea purpurea*; significant reduction of newly formed blood vessels 1.2 and 0.6 mg daily.	2016	Radomska-Lesniewska et al. [331]

* Species not specified; however, all specific attribution must be cautioned against due to the frequent admixture.

**Table 6:** Ex vivo experiments regarding analgesic/antinociceptive and anti-inflammatory effects of devil’s claw preparations and compounds.

Study	Year	Reference
Human whole-blood anticoagulated with heparin; preincubated with Harpagophytum * extract or purified harpagoside; both dose-dependently inhibited cysteinyl-leukotriene and thromboxane B2 release after biotransformation.	1996, 1997	Tippler et al. [332,333]
Human whole-blood assay (healthy and osteoarthritic) for COX-1 and COX-2 activity and NO production; *H. procumbens* extract and harpagoside; increased the activity of baseline COX-1 and COX-2 without LPS, crude extract did not alter COX activity; harpagoside inhibited COX-1, COX-2, and NO.	2007	Anauate [334]
Freshly excised porcine skin; dermal and transcutaneous delivery and effect on COX-2 expression in Western blotting and immunocytochemical assays; *Harpagophytum* extract in various vehicles, harpagoside, harpagide, 8-coumaroylharpagide, and verbascoside; ratio-dependent inhibition of COX-2 expression, higher penetration of all compounds from ethanol/water.	2008	Abdelouahab and Heard [335,336]
Freshly excised porcine skin; transcutaneous delivery and effect on COX-2, PGE2, 5-LOX, and inducible NO synthase (iNOS) expression in Western blotting and immunocytochemical assays; commercial *Harpagophytum* extracts, harpagoside, harpagide, 8-coumaroylharpagide, and verbascoside; ratio-dependent inhibition of COX-2 expression and PGE2, no significant effect on 5-LOX and iNOS, relative proportions of anti- and pro-inflammatory compounds in commercial products varied.	2009, 2010	Ouitas and Heard [337,338,339]
LPS-stimulated human whole-blood assay (healthy) for COX-1 and COX-2 activity and NO production, incubation of isolated fractions obtained by flash chromatography monitored with HPLC, TLC, and identified by _1_HNMR; fractions of *H. procumbens* extract; highest concentration of harpagoside inhibited COX-1, COX-2, and NO; iridoid pool increased COX-2 while NO and COX-1 activities remained unchanged, fraction containing cinnamic acid reduced NO only.	2010	Anaute et al. [340]

* Species not specified; however, all specific attribution must be cautioned against due to the frequent admixture.

**Table 7:** Mixed experiments regarding analgesic/antinociceptive and anti-inflammatory effects of devil’s claw preparations and compounds.

Study	Type	Year	Reference
Carrageenan-induced rat paw edema and adjuvant-induced arthritis in rats; arachidonic acid and prostaglandin synthetase incubated with various concentrations of indomethacin, acetylsalicylic acid, or *Harpagophytum* extract (not specified); no effect on edema, anti-inflammatory activity is not mediated by the inhibition of the prostaglandin synthetase.	In Vitro and in vivo	1983	Whitehouse et al. [341]
Cultured human mammary epithelial cells and female ICR mice; TPA-induced COX expression; *Harpagophytum* methanolic extract (10, 5, 1 µg/mL, 600, 300, 60 µg, respectively); inhibition of COX-2 expression in both models.	In Vitro and in vivo	2004	Na et al. [342]
Rat adjuvant-induced chronic arthritis model, LPS-stimulated mouse macrophage cells (RAW 264.7); *Harpagophytum* ethanolic extract; significant anti-inflammatory effect, and dose-dependent suppression of, IL-6 and TNF-α, respectively.	In Vitro and in vivo	2010	Inaba et al. [343]
Molecular docking study of harpagoside and harpagide with COX-2; binding energies were −9.13 and −5.53 kcal/mol respectively, finding both harpagoside and harpagide to be highly selective COX-2 inhibitors.	Simulation	2016	Rahimi et al. [344]
Mouse myoblast C2C12, human colorectal adenocarcinoma HCT116 cell lines, isolated rat colon challenged with LPS; aqueous *Harpagophytum* extract (1–1000 μg/mL); HCT116 viability reduced, ROS production in both cell lines reduced, PGE2, 8-iso-PGF_2α_, serotonin, and TNF-α production inhibited.	In Vitro and ex vivo	2017	Locatelli et al. [345], Leporini et al. [346]
Antioxidant capacity, leukocyte ROS production, COX-2/PGE2 pathway or cytokine secretions; *H. procumbens* methanolic extract; decreased the secretion of IL-21 and IL-23, increased TNF-α, IL-8, and IFN-γ, immune-stimulant effect.	In Vitro and ex vivo	2019	Cholet et al. [347]
LPS-stimulated wild-type (C57/BL6) male mice colon and HCT116 cells; experimental model of inflammatory bowel disease; *H. procumbens* aqueous extract; anti-inflammatory, antioxidative, and antimicrobial effects (against pathogen fungal strains), morphological alterations in the colon tissue indicated.	In Vitro and ex vivo	2020	Recinella et al. [348]

* Species not specified; however, all specific attribution must be cautioned against due to the frequent admixture.

**Table 8:** Anti-inflammatory targets of *Harpagophytum* preparations and compounds.

		IC_50_							Reference
Extract/Fraction	Harpagoside (%)	Cys-LT	TXB_2_	Enzyme Inhibitors	IL-6	IL-1β	NF-κB	COX-2	
Special extract WS1531	7.3	9.2 µM/L	55.3 µM/L						[332,333]
	7.3	62 µg/mL	373 µg/mL						[285]
Aqueous ethanolic *H. procumbens* extract	2.1	1450 µg/mL	-						[285]
				542 μg/mL (HNE)					[199,201]
				547.69/601.49 µg/mL (MPO) *					[349]
					<100 µg/mL				[297]
*H. procumbens* tincture				915.55/776.49 µg/mL (MPO) *					[349]
Aqueous ethanolic *H. zeyheri* extract				1012 µg/mL (HNE)					[199,201]
Aqueous *H. procumbens* extract	8.9					0.55 µg/mL			[350]
	27					0.2 µg/mL			[350]
Ethanolic *H. procumbens* extract				65.5 µg/mL (FAAH)					[304]
Ethyl acetate fraction of aqueous ethanolic *H. procumbens* extract	19.95	391 µg/mL	-						[285]
Butanol fraction of aqueous ethanolic *H. procumbens* extract	19.5	565 µg/mL	203 µg/mL						[285]
Methanolic *H. procumbens extract*								1046 µg/mL	[295]
*H. procumbens* extracts and isolates								~125 µg/mL	[296]
**Isolated compounds**									
Harpagoside		30 µM/L	48.6 µM/L						[332,333]
		39 μM/L	49 μM/L						[285]
								1041 µg/mL	[295]
				>600 µg/mL (HNE)					[199,201]
				92.7 µM (AChE)					[351]
				95.6 µM (AChE)					[351]
							96.4 µM		[293]
					14.04 µM				[302]
Harpagide								1186 µg/mL	[295]
8-PCHG				179 µg/mL (HNE)					[199,201]
				95.6 µM (AChE)					[351]
Pagoside				154 µg/mL (HNE)					[199,201]
Caffeic acid				86 µg/mL (HNE)					[199,201]
Acetoside				>500 µg/mL (HNE)					[199,201]
				19.9 µM (AChE), 35 µM (BChE)					[351]
Isoacetocide				179 µg/mL (HNE)					[199,201]
				21.6 µM (AChE), 29.7 µM (BChE)					[351]
Decaffeoylverbascoside				16.1 µM (AChE), 46 µM (BChE)					[351]
6′-O-Acetylacteosid				47 µg/mL (HNE)					[199,201]

* Formyl methionyl leucine phenylalanine- and arachidonic acid-stimulated, respectively.

**Table 9:** Experiments regarding other effects of devil’s claw preparations and compounds.

Effect	Study	Type	Year	Reference
Antioxidant	Rats, *Harpagophytum* * extract, 100 and 200 mg/kg bw or selegiline i.p. for 1, 7, or 14 days; dose-dependent increase of superoxide dismutase, catalase, and glutathione peroxidase activities and reduction of lipid peroxidase similar to selegiline after 7 days.	In Vivo	1998	Bhattacharya and Bhattacharya [352]
	Luminol-enhanced chemiluminescence in a xanthine/xanthine oxidase cell-free system; *Harpagophytum* root powder; superoxide and peroxyl were scavenged dose-dependently.	In Vitro	2002	Langmead et al. [353]
	Trolox equivalent antioxidant capacity (TEAC) assay; *Harpagophytum* aqueous extract (2.6% harpagoside) and harpagoside; extract rich in water-soluble antioxidants, harpagoside showed poor activity.	In Vitro	2003	Betancor-Fernandez et al. [354]
	Rat renal mesangial cells; IL-1β-induced NO formation and transcriptional regulation of iNOS; *H. procumbens* extracts with varying harpagoside content and pure harpagoside; dose-dependent and harpagoside-independent inhibition of iNOS expression.	In Vitro	2004	Kaszkin et al. [350]
	*Harpagophytum* aqueous extract; protection from DNA-damaging effects of stannous chloride in proficient and deficient *E. coli* model; possible chelating, scavenger, or oxidant activity postulated.	In Vitro	2007	Almeida et al. [355]
	Antioxidant characteristics using in vitro test systems, DPPH radical scavenging, stimulated nitrite generation, neutrophil superoxide anion generation, and neutrophil myeloperoxidase (MPO); *Harpagophytum* extract (1.2% harpagoside), tincture, harpagoside; dose-dependent effect in all models, minimal scavenging activity of harpagoside.	In Vitro	2005, 2009	Grant et al. [349], Grant [356]
	Antioxidant activities of total methanol extracts, fractions (phenylethanoids, terpenoids, and sugars), and β-OH-verbascoside, verbascoside, and leucosceptoside from cell suspension culture of *H. procumbens*; DPPH, superoxide anion generation, and oxygen radical absorbance capacity (ORAC) assays; β-OH-verbascoside most active in DPPH and superoxide anion generation, leucosceptoside in ORAC.	In Vitro	2010	Georgiev et al. [357]
	Ferric-reducing antioxidant power test; *H. procumbens* crude methanolic extract, phenylethanoid-containing fraction, and verbascoside; strong ferrous ion-chelating capacity.	In Vitro	2011	Georgiev et al. [358]
	Brain homogenates, catalase activity and thiol levels, brain cortical slices; lipid peroxidation, antioxidant defenses, cell damage, respectively; *H. procumbens* infusion, crude extract, and fractions; dose-dependent inhibition of lipid peroxidation, ethyl acetate fraction had the highest antioxidant effects.	In Vitro	2013	Schaffer et al. [359,360]
	Human neutrophils challenged with phorbol myristate acetate (PMA), opsonized *Staphylococcus aureus*, and *Fusobacterium nucleatum*; 5 taxa of *Harpagophytum*, including one hybrid; high variability in suppression of respiratory burst, hybrid with highest antioxidant capacity but proinflammatory effect, three taxa with anti-inflammatory effect.	In Vitro	2016	Muzila et al. [361]
	Adult male Wistar rats, fluphenazine-induced orofacial dyskinesia (OD); DPPH assay; ethyl acetate fraction of *H. procumbens* (10, 30, or 100 mg/kg i.p.); inhibition of vacuous chewing movements, decreased locomotion unchanged, protective against change in catalase activity, not against ROS increase.	In Vivo	2016	Schaffer et al. [362]
	Porcine neutrophils; respiratory burst; harpagoside; significant inhibition of ROS production.	In Vitro	2017	Mosca et al. [363]
	Male Sprague–Dawley rats, modified rodent contusion model of spinal cord injury, murine BV-2 microglial cells; *H. procumbens* hydroethanolic extract (5.3% harpagoside, 300 mg/kg); behavioral and neurochemical parameters, improved, some significantly, in cell line, oxidative stress and inflammatory response were suppressed.	In Vitro and in vivo	2020	Ungerer et al. [364]
	LPS-induced RAW 264.7 mouse and U937 human macrophages; DPPH and 2,2′-azino-bis (3-ethylbenzothiazoline-6-sulphonic acid) (ABTS) assays; aqueous, ethanolic, and ethyl acetate extracts of *H. zeyheri*; for all extracts, dose-dependent inhibition of IL-10 expression, ethyl acetate fraction with lowest IC_50_ in both assays, NO and TNF-α inhibition similar to diclofenac.	In Vitro	2021	Ncube et al. [365]
Antidiabetic	Streptozotocin-induced diabetes mellitus in rats; *H. procumbens* root aqueous extract (50–800 mg/kg i.p.); significant reduction in blood glucose levels in normal and diabetic rats.	In Vitro	2004	Mahomed and Ojewole [317], Mahomed [318]
Anticholinesterase	Chick, guineapig, and rabbit isolated gastro-intestinal smooth muscle preparations; *H. procumbens* root aqueous extract (10–1000 µg/mL); dose-dependent contractions of gastro-intestinal tract smooth muscles.	In Vitro	2005	Mahomed [318], Mahomed et al. [366]
	Spectrophotometric method using acetylthiocholine and butyrylcholine chloride as substrates; *H. procumbens* crude methanolic extract, phenylethanoid-containing fraction, and verbascoside; significant cholinesterase inhibitory activity.	In Vitro	2011	Georgiev et al. [358]
	Spectrophotometric method, acetylcholinesterase (AchE) and butyrylcholinesterase (BchE) inhibition; *H. procumbens* ethyl acetate extract and fractions; inhibition by verbascosides > 60%	In Vitro	2013	Bae et al. [351]
Antimicrobial	*Harpagophytum* extract (not specified) showed mild antifungal effects against *Penicillum digitatum* and *Botrytis cinerea*.	In Vitro	1985	Guérin and Réveillère [367]
	*Harpagophytum* dry extract (2.6% harpagoside) and harpagoside; inhibition of a panel (all) of aerobic bacteria, *C. krusei,* and two anaerobic bacteria strains, harpagoside without effect.	In Vitro	2007	Weckesser et al. [368]
	Chloroquine (CQ)-sensitive and CQ-resistant strains of *P. falciparum*, and cytotoxicity in CHO and HepG2 cells; extracts of *H. procumbens* aerial parts and seeds, and petrol ether of the root, (+)-8,11,13-totaratriene-12,13-diol and ferruginol, and CQ diphosphate as control; the two diterpenes showed significant inhibition of both strains without being cytotoxic.	In Vitro	2003	Clarkson et al. [200]
	Female Balb/c mice, infected with *Toxocara canis*; *Harpagophytum* ethanolic extract (100 mg/kg); decrease in eosinophil accumulation, IL-5 and IgE significantly decreased.	In Vivo	2012, 2014	Oliveira et al. [369,370,371]
	*Harpagophytum* ethanolic extract showed dose-dependent effect on *Schistosoma mansoni*, mechanism of action proposed; proteins relevant for cellular homeostasis identified as possible targets.	In Vitro	2014	Correia [372]
	Bacterial triggers of rheumatoid arthritis, ankylosing spondylitis, multiple sclerosis, and rheumatic fever; powdered *Harpagophytum* extracts, various solvents; inhibition of *Proteus mirabilis*, *Klebsiella pneumoniae*, *Acinetobacter baylyi*, *Pseudomonas aeruginosa,* and *Streptococcus pyogenes* throughout, methanolic extract more potent, no toxicity in *Artemia nauplii* bioassay. (Note: throughout the publication, the substance of investigation is mislabeled as devil’s claw *fruit*, while it was, in fact, the root being investigated (pers. comm. Ian Cock, 2021))	In Vitro	2017	Cock and Bromley [373]
Antimutagenic	Cultured human lymphocytes; mutagenic activity of 1-nitropyrene (1-Npy) in cytokinesis-block micronucleus assay; *Harpagophytum* aqueous-ethanolic extract, harpagoside; genotoxicity significantly reduced for both, only harpagoside significantly reduced the mutagenicity of 1-Npy.	In Vitro	2014, 2015	Luigi [374], Luigi et al. [375]
Anti-osteoporotic	Male ICR mice, female C57BL/6J mice; receptor activator of nuclear factor κ-Β ligand (RANKL)-induced osteoclastogenesis; harpagoside; inhibition of RANKL, osteoclast formation, and LPS-induced bone loss, but not ovariectomy-mediated bone erosion.	In Vitro	2015	Kim et al. [376]
	Mouse calvaria MC3T3-E1cells; bone formation and resorption, bone-loss in ovariectomized (OVX) mouse model; harpagide; stimulated differentiation and maturation of osteoblast cells and suppressed RANKL-induced osteoclastogenesis, improved bone recovery in OVX model, inhibited markers of bone loss in the serum.	In Vitro and in vivo	2016	Chung et al. [377]
	Mouse calvaria MC3T3-E1cells; bone formation and resorption, bone-loss in ovariectomized (OVX) mouse model; harpagoside; stimulated differentiation and maturation of osteoblast cells and suppressed RANKL-induced osteoclastogenesis, improved bone recovery in OVX model, inhibited markers of bone loss in the serum.	In Vitro and in vivo	2017	Chung et al. [378]
Cardiovascular	Frog and guineapig hearts, cats; cardiac muscle contraction and blood pressure, dose-dependent positive and negative inotropic effects, no effect on blood pressure.	In Vitro and in vivo	1965	Vollmann [379]
	Normotensive rats, rabbit heart; methanolic extract of *Harpagophytum*, harpagoside, harpagide; decrease in blood pressure and heart rate observed, less with harpagoside; extract mild inotropic at lower and negative inotropic at higher doses, harpagoside more negative chronotropic and positive inotropic, harpagide only slightly negative chronotropic but considerably negative inotropic.	In Vitro and in vivo	1984	Circosta et al. [380]
	Rat heart; methanolic extract of *Harpagophytum* (8.5% harpagoside and 10.5% total iridoids) and harpagoside; significant, dose-dependent, protective action toward hyperkinetic ventricular arrhythmias.	In Vitro	1985	De Pasquale et al. [381]
	Langendorff preparations of rat heart; ischemic perfusion induced hyperkinetic ventricular arrhythmia; *H. procumbens*, harpagoside; significant, dose-dependent protective action for both.	In Vitro	1985	De Pasquale et al. [382]
	Guineapig ileum and rabbit jejunum; *Harpagophytum* extract, harpagoside, harpagide; spasmolytic effect, strongest for harpagoside.	In Vitro	1985	Occhiuto et al. [383]
	Dogs; harpagoside, harpagide (3.4 mg/kg); decrease of mean aortic pressure with harpagoside.	In Vivo	1990	Occhiuto and de Pasquale [384]
	Multiple mammalian animal models; *H. procumbens* root aqueous extract (10–400 mg/kg i.v., 10–1000 µg/mL); dose-dependent, significant hypotensive, cardio-depressant, and vasorelaxant effects.	In Vitro and in vivo	2004	Mahomed and Ojewole [385], Mahomed [318]
Neuroprotective	Pentylenetetrazole (PTZ)-, picrotoxin (PCT)-, and bicuculline (BCL)-induced seizures in mice; *H. procumbens* aqueous extract (100–800 mg/kg i.p.); PZT-induced seizures significantly reduced, PCT and BCL to a lesser extent, CNS depressed.	In Vivo	2006	Mahomed and Ojewole [386]
	Rat hypothalamic (Hypo-E22) cells and rat cortex challenged with amyloid β-peptide; *H. procumbens* aqueous extract; increased brain-derived neurotrophic factor gene expression and decreased TNF-α gene expression in Hypo-E22 cells, alleviated decreased monoaminergic signaling in cortex presynaptic endings.	In Vitro and ex vivo	2017	Ferrante et al. [387]
	Male Wistar rats; chronic cerebral hypoperfusion model; harpagoside (15 mg/kg, 60 days); symptoms of vascular dementia spatial and fear memory impairments restored, phosphatase and tensin homolog (PTEN) significantly suppressed.	In Vivo	2018	Chen et al. [388]
	Female Wistar albino rats, arsenic induced neurotoxicity; *Harpagophytum* powder (200 and 400 mg/kg, p.o.); behavioral and biochemical parameters improved significantly.	In Vivo	2020	Peruru et al. [389]
Immunomodulatory/thymomimetic	Maturation of mice thymocytes in the presence of a glycocorticosteroid, cytotoxicity by microscopy and flow cytometry; ethanolic extract of *Harpagophytum*, *Filipendula ulmaria*, and *Echinacea purpurea*, various dilutions; 17% increase in the number of surviving cells.	In Vitro	2002	Prosinska et al. [390]
Anorexigenic	Male C57BL/6 mice, calcium mobilization and growth hormone secretagogue receptor (GHS-R1a) internalization; *Harpagophytum* root powder; significantly increased cellular calcium influx but no induction of GHS-R1a receptor internalization, significant anorexigenic effect.	In Vivo	2014	Torres-Fuentes et al. [391]
	Male Wistar rats; obestatin secretion; *Harpagophytum* hydroalcoholic extract (150, 300, and 600 mg/kg); significantly increased serum levels of obestatin and reduced body weight at 300 and 600 mg/kg.	In Vivo	2016	Saleh et al. [392]
Metal accumulation	Rats, supplemented with lead acetate; *Harpagophytum* infusion (30 mg/kg); significant reduction of lead deposits	In Vitro	1975	Int. Bio Research [393]

* Species not specified; however, all specific attribution must be cautioned against due to the frequent admixture.

**Table 12:** Patents pertaining to *Harpagophytum* and its preparations.

Title	Date	Number
Food supplement	4/3/1984	US19810287235
Therapeutically active mixture	11/8/1984	DE19833316726
Homeopathic remedy for the treatment of rheumatic disorders	11/19/1987	DE19863616054
Plant-based medicinal composition for internal use	4/22/1988	FR19860014608
Medicinal combination based on plants and trace elements for the treatment of rheumatism and inflammatory states	11/10/1988	FR19870006450
Process for the preparation, by extracting, of *Harpagophytum*	7/13/1992	KR19890016112
Anti-pruritic cosmetic composition containing *Harpagophytum* root extract	1/27/1993	EP19920402100
Preparation of concentrated plant extract, particularly from *Harpagophytum procumbens*	8/7/1997	DE1996103788
Harpagosid-angereicherter Extrakt aus *Harpagophytum procumbens* und Verfahren zu seiner Herstellung [harpagoside- enriched extract of ***H. procumbens*** and its manufacture]	10/2/1997	DE1996151290
A purified extract from *Harpagophytum procumbens* and/or *Harpagophytum zeyheri*, a process for its preparation and its use	12/18/1997	Google
Skin care composition contains peroxidized fatty substance, e.g., unsaturated vegetable oils and plant extract	3/20/1998	FR19960011438
Natural composition for treating bone or joint inflammation	11/26/1998	WO1998US10758
Micro-nutritional compositions having a therapeutic effect containing polyunsaturated fatty acids, trace elements, and vitamins	7/16/1999	FR19980000331
A method of producing high anti-inflammatory activity extracts from *Harpagophytum procumbens*	10/6/1999	GB19980006971
Effervescent preparation containing a plant extract	6/16/1999	EP0922450A1
Method for producing high activity extracts from *Harpagophytum procumbens*	3/6/2001	US19990280499
Harpagoside-enriched extract from *Harpagophytum procumbens* and processes for producing the same	8/28/2001	US19990155043
Dietary supplement	12/18/2001	JP20000172296
Pharmaceutical preparation containing *Cibotii rhizoma* and *Harpagophytum procumbens* DC extracts as main ingredients	6/3/2002	KR20000071397
Skin care preparation	6/4/2002	JP20000402968
Pharmaceutical composition with anti-atherosclerotic activity	6/5/2002	EP20010128629
Use of harpagide-related compound as prophylactic and therapeutic agent of osteoporosis, arthritis, and disc and pharmaceutical composition containing compound as effective ingredient	11/16/2002	KR20000071497
Composition useful for treating or preventing osteoarthritis, especially in horses, containing extract(s) of Equisetum arvense, *Symphytum officinale,* and/or *Harpagophytum procumbens*	3/27/2003	DE2001143146
Use of active substance mixtures containing tocopherols and *Harpagophytum procumbens* extracts for the preparation of a drug against rheumatic arthritis	12/17/2003	EP20020012765
Chewing gum composition with vegetal additives	7/29/2004	WO2003EP14600
Pain-relieving agent containing extract of *Harpagophytum procumbens*, *Corydalis turtschanovii,* and *Atractylodes japonica*	2/5/2005	KR20030052489
Treating or preventing renal diseases, dysfunction, and/or damage, e.g., degenerative and/or inflammatory renal disease, using *Harpagophytum extract* or harpagoside	3/10/2005	DE2003126556
Phyto-composition for the treatment of articular diseases		WO2005092355
Use of devil’s claw (*Harpagophytum procumbens*) root extracts for endometriosis treatment	11/2/2006	WO2006EP61831
A method for separating harpagide from *Harpagophytum procumbens*	2/5/2007	KR20050102609
Activator of peroxisome proliferator-activated receptor (PPAR)	5/17/2007	JP20050317156
Adjuvant composition for physiotherapy	7/24/2007	KR20060005183
Maillard reaction inhibitor, skin care preparation containing the same, and food and beverage	10/4/2007	JP20060080104
Phyto-composition for the treatment of joint diseases	12/13/2007	US20050594439
Natural remedy–dietary supplement combination product	9/4/2008	US20060815432
Root extract of *Harpagophytum* for stimulating hair growth	5/27/2009	EP20070802633
Skin care preparation, oral composition, and food and drink	10/22/2009	JP20080091677
Novel method for preparing purified extracts of *Harpagophytum procumbens*	12/9/2010	US20080599146
Animal food compositions	7/21/2011	WO2010US60804
Compositions comprising plant extracts and use thereof for treating inflammation	10/27/2011	US200913120739
Anti-inflammatory composition	12/21/2011	EP20110170436
Antirheumatic body cream composition	12/30/2011	RO20110000644
Pharmaceutical composition for preventing and treating metabolic bone disease comprising of *Harpagophytum*	6/18/2012	KR20110147135
Phyto-concentrated composition, useful as antispasmodic relaxant, and muscular comfort to, e.g., enhance relaxation of painfully contracted muscle tissue, comprises, e.g., cannabis sativa and an excipient comprising, e.g., castor oil	10/12/2012	FR20110001030
Nonabrasive toothpaste containing enzyme papain, *Harpagophytum* extract d,l-pyrrolidone carboxylate n-cocoyl ethyl arginate, and sodium fluoride	7/20/2013	RU20120101119
Cosmetic composition for calming and applying an electric current of skins and manufacturing the same	12/27/2013	KR20120065152
Anti-rheumatism medicinal liquor and preparation method thereof	3/19/2014	CN20131645408
Composition containing chondroitin sulfate and hyaluronidase	12/10/2014	RU20130123301
Mucoadhesive devil’s claw extracts (*Harpagophytum procumbens*) and uses thereof	3/11/2015	EP20140184267
Compositions for alleviating, preventing, or treating pain comprising *Harpagophytum procumbens* and *Acanthopanax senticosus* extracts as active ingredients	6/8/2015	KR20130146128
Traditional Chinese medicine composite for treating gout	7/8/2015	CN20151209743
Cell line cultures from plants belonging to the *Harpagophytum* genus	1/4/2018	WO2017EP65814
Method for preparing purified extracts of *Harpagophytum procumbens*	30/10/2018	US20100311675A1
Oral herbal pain killer formulations	15/10/2020	WO2020208395A1
Polyherbal transdermal patch for pain management and its process of preparation	22/10/2020	WO2020212820A2
External medicine for inhibiting postoperative venous thrombosis and application thereof	19/2/2021	CN109589331B
Freedom (nutritional supplement)	9/2/2021	US20200060320A1

## Data Availability

All data has been presented in the main text.
